# Parathyroid hormone signaling in mature osteoblasts/osteocytes protects mice from age-related bone loss

**DOI:** 10.18632/aging.203808

**Published:** 2021-12-30

**Authors:** Yuhei Uda, Vaibhav Saini, Christopher A. Petty, Majed Alshehri, Chao Shi, Jordan M. Spatz, Roberto Santos, Carly M. Newell, Tim Y. Huang, Alejandro Kochen, Ji W. Kim, Christodoulos K. Constantinou, Hiroaki Saito, Kathryn D. Held, Eric Hesse, Paola Divieti Pajevic

**Affiliations:** 1 Department of Translational Dental Medicine, Goldman School of Dental Medicine, Boston University, Boston, MA 02118, USA; 2 Endocrine Unit, Massachusetts General Hospital, Harvard Medical School, Boston, MA 02114, USA; 3 Department of Orthopaedics, The Second Affiliated Hospital of Xi’an Jiaotong University, Xi’an 710004, Shaanxi Province, P.R. China; 4 School of Medicine, University of California San Francisco, San Francisco, CA 94143, USA; 5 Heisenberg-Group for Molecular Skeletal Biology, University Medical Center Hamburg-Eppendorf, Hamburg 20251, Germany; 6 Radiation Oncology, Massachusetts General Hospital, Harvard Medical School, Boston, MA 02114, USA

**Keywords:** osteocyte, oxidative stress, aging, osteoporosis, parathyroid hormone

## Abstract

Aging is accompanied by osteopenia, characterized by reduced bone formation and increased bone resorption. Osteocytes, the terminally differentiated osteoblasts, are regulators of bone homeostasis, and parathyroid hormone (PTH) receptor (PPR) signaling in mature osteoblasts/osteocytes is essential for PTH-driven anabolic and catabolic skeletal responses. However, the role of PPR signaling in those cells during aging has not been investigated. The aim of this study was to analyze the role of PTH signaling in mature osteoblasts/osteocytes during aging. Mice lacking PPR in osteocyte (Dmp1-PPR^KO^) display an age-dependent osteopenia characterized by a significant decrease in osteoblast activity and increase in osteoclast number and activity. At the molecular level, the absence of PPR signaling in mature osteoblasts/osteocytes is associated with an increase in serum sclerostin and a significant increase in osteocytes expressing 4-hydroxy-2-nonenals, a marker of oxidative stress. In Dmp1-PPR^KO^ mice there was an age-dependent increase in p16^Ink4a^/*Cdkn2a* expression, whereas it was unchanged in controls. *In vitro* studies demonstrated that PTH protects osteocytes from oxidative stress-induced cell death. In summary, we reported that PPR signaling in osteocytes is important for protecting the skeleton from age-induced bone loss by restraining osteoclast’s activity and protecting osteocytes from oxidative stresses.

## INTRODUCTION

Osteoporosis affects an estimated 200 million people worldwide and it becomes increasingly prevalent in the aging population [1, 2]. It is well established that the first 34 amino acids of PTH and PTH-related peptide (PTHrP) are necessary and sufficient to fully activate the PTH/PTHrP receptor (PPR) and both PTH (teriparatide) and PTHrP (Abaloparatide) are approved anabolic agents to treat osteoporosis. PPR is coupled to G-proteins capable of activating multiple pathways, including those signaling through cyclic adenosine monophosphate (cAMP)/protein kinase A (PKA), phospholipase C (PLC)/protein kinase C (PKC), and non-PLC-dependent PKC and Ca_i_^++^ [3]. In the skeleton, PTH and PTHrP exert their anabolic and catabolic effects by binding and activating the PPR expressed on cells of the osteoblast lineage. This lineage comprises a variety of cells, from osteoprogenitors to mature osteoblasts and osteocytes; however, cellular targets of PTH actions are still not completely understood.

Osteocytes, the terminally differentiated osteoblasts deeply embedded in the bone mineral matrix, comprise ~95% of all cells in the adult bone [4–6]. Recent literature supports direct and indirect interactions of osteocytes with nearby cells, including osteoblasts, osteoclasts, and endothelial cells and with distant organs, such as kidneys and muscles, through various secreted molecules, including receptor-activator of nuclear factor-κB ligand (RANKL), fibroblast growth factor 23 (FGF23) and sclerostin [7–13]. Sclerostin, a potent Wnt inhibitor, suppresses osteoblast function and proliferation, whereas RANKL is a master regulator of osteoclast differentiation and survival [14, 15]. In addition, recent studies identified osteocytes as critical effectors in normal physiological processes, such as lactation, hematopoiesis, and bone modeling and remodeling [8, 10, 15, 16]. Osteocytes may also play important roles in diseases such as hypophosphatemic rickets, osteopenia, sclerosteosis, Van Buchem disease, and osteopetrosis [17–19].

Mice with constitutively active PPR in osteocytes display increased trabecular bone mass, increased osteoblast number, and decreased *Sost*/sclerostin expression [20, 21] whereas mice lacking RANKL in osteocytes have high bone mineral density and osteopetrosis [14, 15], demonstrating an important role for osteocytes in bone remodeling. We have previously generated mice with conditional knockout (KO) of the PPR predominantly in osteocytes by using the 10-Kb dentin matrix protein 1 (Dmp1) promoter to drive Cre recombinase expression in PPR-floxed mice (Dmp1-PPR^KO^) [22]. At 3months of age, Dmp1-PPR^KO^ mice show normal serum calcium, phosphate, and PTH, suggesting that under physiological conditions PPR signaling in osteocytes is not needed to maintain normal mineral homeostasis. These mice display a significant increase in trabecular and cortical bone, indicating that PPR on osteocytes is required for normal bone remodeling. When subjected to intermittent or continuous PTH administration, Dmp1-PPR^KO^ mice generated blunted anabolic and catabolic skeletal responses, indicating that PPR signaling in osteocytes is necessary for full skeletal responses to the hormone [22].

To study the role of PPR signaling in osteocytes in age-dependent osteopenia, we analyzed the skeletal phenotype of mice at 4 (adult) and 13 (middle-aged) months of age. As compared with controls, 4-month-old Dmp1-PPR^KO^ animals showed increased trabecular bone and decreased osteoclast number and activity, whereas at 13 months these mutant mice had a significant decrease in trabecular bone associated with increased osteoclast number and activity. *In vitro*, PTH significantly protected osteocytic cells from hydrogen peroxide (H_2_O_2_) induced cell death and reactive oxygen species (ROS) production. This effect was lost in cells lacking receptor expression. All together these data highlight an essential role of PPR signaling in osteocytes to protect against age-related bone loss and oxidative stresses.

## RESULTS

### PPR ablation in mature osteoblasts/osteocytes induces severe osteopenia in 13-month-old male mice

**Figure 1:**
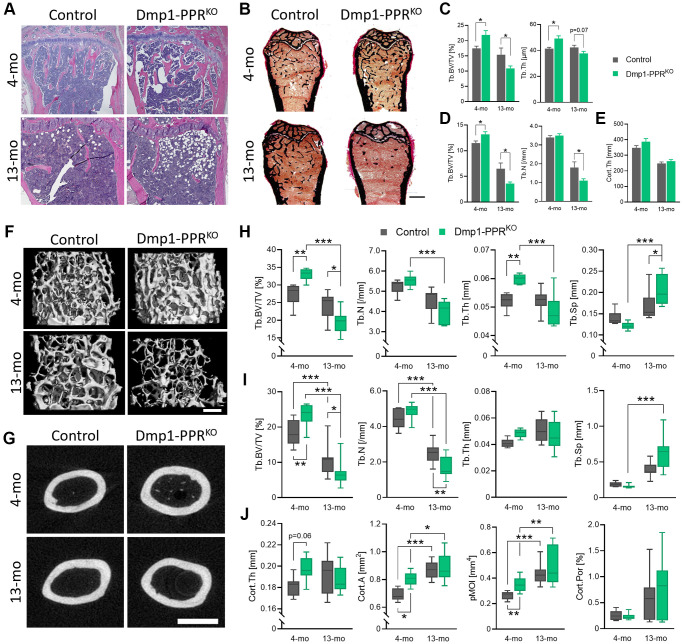
**Age-dependent bone loss in Dmp1-PPR^KO^ mice.** Vertebrae and long bones of male control and KO animals were analyzed by (**A**) histology, (**B**–**E**) histomorphometry and (**F**–**J**) μCT. (**A**) Representative H&E of the proximal tibiae and (**B**) Von Kossa staining of the distal femora. Bar = 1.0 mm. Histomorphometric analysis of (**C**) the L5 and (**D**) trabecular and (**E**) cortical region in the distal and midshaft femora, respectively. *N* = 6–10 per group. Data are presented as mean ± SEM. Representative μCT images of (**F**) the distal and (**G**) the midshaft femora. Bars = (**F**) 300 μm and (**G**) 1.0 mm. μCT analysis of (**H**) the L5 and (**I**) the distal femur (trabecular) and (**J**) the midshaft femur (cortical) are shown. Data are presented as box and whisker plot. *N* = 6–16 per group. See Tables 1 and 2 for the full list of parameters. Analyses were performed in a blinded fashion. Unpaired Student’s *t* test (**C**–**E**) and Two-way ANOVA with Tukey’s *post hoc* test or Mann-Whitney test (**H**–**J**) was performed. ^*^p < 0.05, ^**^p < 0.01, ^***^p < 0.001. Abbreviations: Tb: Trabecular; Cort: cortical; BV: bone volume; TV: total tissue volume; Th: thickness; N: number; Sp: separation; A: area; pMOI: polar moment of inertia; Por: porosity.

Mice lacking PPR in mature osteoblasts/osteocytes, namely Dmp1-PPR^KO^, have increased bone mineral density and bone mass by three months of age and they are resistant to both the anabolic and catabolic effects of PTH [22]. To investigate whether PPR signaling in mature osteoblasts/osteocytes is needed to maintain skeletal homeostasis during age-dependent bone loss, we analyzed the skeletons of adult and middle-aged animals. Hematoxylin and eosin (H&E) staining of the tibiae and von Kossa staining of the fifth lumbar (L5) vertebrae and the femora showed significant increase in trabecular bone volume over total tissue volume (BV/TV%) at 4 months, as previously reported for young mice [22]. The increased bone mass was followed by a dramatic bone loss in Dmp1-PPR^KO^ mice at 13 months, as compared to littermate controls (Figure 1A–1D, Table 1, Supplementary Figure 1A). Cortical thickness was similar between Dmp1-PPR^KO^ and controls at both ages (Figure 1E). Micro-computed tomography (μCT) analysis further confirmed the histological data (Figure 1F–1J, Table 2). At 4 months of age, in Dmp1-PPR^KO^ mice there was a significant increase in BV/TV% in L5 and distal femur (Figure 1H–1I) and a significant increase in trabecular thickness (Figure 1H) in vertebral bones. In contrast, at 13 months of age, BV/TV% in both sites (L5 and femurs) were significantly decreased in Dmp1-PPR^KO^ mice compared to littermate controls (Figure 1H, 1I). The significant reduction in these trabecular parameters was still present in KO animals at 16 months of age (Supplementary Figure 1B). In Dmp1-PPR^KO^ mice, bone volume of L5 and the distal femora decreased by 41% and 71%, respectively, from 4 to 13 months, whereas in control animals this decrease was 12% and 42%, respectively (Figure 1H, 1I). At 13 months of age, trabecular separation was also significantly increased in the L5 of Dmp1-PPR^KO^ mice (Figure 1H) and trabecular number (Tb.N) was significantly decreased in the distal femora (Figure 1I) of Dmp1-PPR^KO^ mice compared to controls. Taken together this data revealed a marked age-dependent trabecular bone loss in the absence of PPR signaling in mature osteoblasts/osteocytes.

**Table 1:** Dynamic histomorphometric analysis of trabecular and cortical bone in 4- and 13-month-old Dmp1-PPR^KO^ and control mice. Trabecular bone parameters measured in L5 vertebrae and distal femurs. Cortical bone parameters measured in midshaft of femurs. Values are expressed as mean ± SEM, two-tailed *t* test assuming equal variance was performed to compare control vs. Dmp1-PPR^KO^ male mice at 4 and 13 months of age. *p* < 0.05 in bold and italics.

**Parameter**	**4-month-old**			**13-month-old**		
	**Control**	**Dmp1-PPR^KO^**	***p* value**	**Control**	**Dmp1-PPR^KO^**	***p* value**
**L5 vertebrae**	***n* = 6**	***n* = 10**		***n* = 8**	***n* = 10**	
*Structural*						
BV/TV (%)	17.5 ± 0.7	21.9 ± 1.4	***0.04***	15.4 ± 2.2	10.9 ± 0.8	***0.04***
Tb.N (/mm)	4.23 ± 0.12	4.42 ± 0.15	0.40	3.56 ± 0.38	2.89 ± 0.17	0.09
Tb.Th (μm)	41.4 ± 0.97	49.2 ± 2.04	***0.01***	42.3 ± 1.71	37.7 ± 1.62	0.07
Tb.Sp (μm)	196.0 ± 7.4	179.3 ± 8.9	0.22	260.3 ± 37.9	321.4 ± 23.8	0.17
**Distal femurs**	***n* = 6**	***n* = 7**		***n* = 8**	***n* = 10**	
*Structural*						
BV/TV (%)	11.5 ± 0.4	13.2 ± 0.5	***0.02***	6.5 ± 1.1	3.6 ± 0.3	***0.04***
Tb.N (/mm)	3.4 ± 0.1	3.5 ± 0.1	0.83	1.8 ± 0.3	1.1 ± 0.1	***0.04***
Tb.Th (μm)	33.5 ± 0.4	38.2 ± 1.6	***0.02***	35.5 ± 1.4	34.6 ± 2.0	0.72
Tb.Sp (μm)	260.0 ± 9.7	252.7 ± 6.4	0.55	665.2 ± 128.5	959.8 ± 65.3	0.08
*Dynamic*						
MS/BS (%)	33.5 ± 0.8	27.38 ± 1.9	***0.04***	21.8 ± 1.5	22.7 ± 1.5	0.07
MAR (μm/day)	1.90 ± 0.2	1.72 ± 0.1	0.33	1.56 ± 0.3	0.95 ± 0.1	***0.02***
BFR/BS	231.5 ± 13.8	173.9 ± 18.5	0.06	130.8 ± 27.7	79.1 ± 8.3	0.71
BFR/BV (%/year)	1320.7 ± 95.0	840.1 ± 67.9	***<0.01***	650.3 ± 109.9	478.4 ± 55.8	0.07
BFR/TV (%/year)	159.8 ± 8.4	116.2 ± 13.1	***0.04***	55.7 ± 17.4	16.1 ± 1.6	0.13
*Formation*						
OV/BV (%)	0.35 ± 0.11	0.28 ± 0.03	0.45	0.51 ± 0.08	0.42 ± 0.06	0.42
OS/BS (%)	6.05 ± 1.25	5.09 ± 0.34	0.38	4.68 ± 0.77	3.32 ± 0.37	0.17
Ob.S/BS (%)	6.36 ± 1.18	5.14 ± 0.37	0.25	5.40 ± 0.88	3.78 ± 0.48	0.16
N.Ob/T.Ar	41.29 ± 6.54	31.58 ± 2.06	0.11	14.26 ± 3.86	5.69 ± 0.87	0.08
N.Ob/B.Pm (/mm)	6.04 ± 0.58	5.39 ± 0.68	0.52	3.83 ± 0.71	2.47 ± 0.31	0.13
*Resorption*						
ES/BS (%)	1.81 ± 0.30	0.97 ± 0.18	***0.04***	0.50 ± 0.13	1.25 ± 0.21	**0.01**
Oc.S/BS (%)	1.62 ± 0.27	0.83 ± 0.15	***0.01***	0.45 ± 0.11	1.24 ± 0.23	**0.01**
N.Oc/B.Pm (/mm)	0.57 ± 0.09	0.34 ± 0.07	0.05	0.25 ± 0.07	0.65 ± 0.13	**0.03**
*Osteocyte*						
N.Ot/BV (/mm^2^)	592.0 ± 59.1	590.9 ± 39.2	0.99	388.1 ± 43.2	300.0 ± 47.1	0.11
**Midshaft femurs**	***n* = 6**	***n* = 10**		***n* = 8**	***n* = 10**	
*Structural*						
Cort.Th (mm)	347.9 ± 14.1	386.9 ± 20.2	0.14	247.5 ± 9.7	262.0 ± 10.4	0.18
*Dynamic*						
End.Cort MAR (mm/day)	1.8 ± 0.1	1.8 ± 0.2	0.84	1.5 ± 0.1	1.2 ± 0.1	0.09
*Osteocyte*						
Ot density (/mm^2^)	470.7 ± 26.8	379.9 ± 25.6	***0.03***	445.3 ± 21.0	421.5 ± 18.1	0.23

**Table 2:** μCT analysis of trabecular and cortical bone in 4- and 13-month-old Dmp1-PPR^KO^ and control. Trabecular bone parameters measured in L5 vertebrae and distal femurs. Cortical bone parameters measured in midshaft of femurs. Values are presented as mean ± SEM, two-tailed *t* test assuming equal variance was performed to compare control vs. Dmp1-PPR^KO^ male mice at 4- and 13-months of age. *p* < 0.05 in bold and italics.

**Parameter**	**4-month-old**			**13-month-old**		
	**Control**	**Dmp1-PPR^KO^**	***p* value**	**Control**	**Dmp1-PPR^KO^**	***p* value**
**L5 vertebrae**	***n* = 6**	***n* = 10**		***n* = 7**	***n* = 11**	
BV/TV (%)	27.4 ± 1.3	33.0 ± 0.5	***<0.001***	24.1 ± 1.5	19.4 ± 0.9	***0.011***
Tb.N (/mm)	5.24 ± 0.149	5.52 ± 0.087	0.101	4.59 ± 0.23	4.01 ± 0.16	0.052
Tb.Th (mm)	0.052 ± 0.001	0.060 ± 0.001	***<0.001***	0.052 ± 0.002	0.049 ± 0.002	0.137
Tb.Sp (mm)	0.14 ± 0.007	0.12 ± 0.003	***0.015***	0.17 ± 0.014	0.21 ± 0.010	0.053
**Distal femurs**	***n* = 7**	***n* = 10**		***n* = 12**	***n* = 15**	
BV/TV (%)	18.0 ± 1.4	23.6 ± 1.0	***0.003***	10.4 ± 1.1	6.8 ± 0.7	***0.013***
Tb.N (/mm)	4.38 ± 0.2	4.87 ± 0.1	0.056	2.46 ± 0.2	1.69 ± 0.2	***0.002***
Tb.Th (mm)	0.041 ± 0.001	0.048 ± 0.001	***<0.001***	0.051 ± 0.003	0.047 ± 0.003	0.285
Tb.Sp (mm)	0.19 ± 0.01	0.16 ± 0.01	***0.028***	0.39 ± 0.03	0.62 ± 0.06	***0.002***
**Midshaft femurs**	***n* = 7**	***n* = 10**		***n* = 12**	***n* = 16**	
Cort.Th (mm)	0.181 ± 0.004	0.197 ± 0.003	***0.005***	0.193 ± 0.005	0.187 ± 0.003	0.30
Cort.Dens (mmHA/ccm)	1239.8 ± 3.9	1218.6 ± 4.4	***0.004***	1288.5 ± 9.5	1279.4 ± 7.1	0.44
Cort.A (mm^2^)	0.69 ± 0.02	0.81 ± 0.02	***<0.001***	0.87 ± 0.02	0.89 ± 0.02	0.50
MA (mm^2^)	0.77 ± 0.03	0.89 ± 0.04	***0.040***	0.98 ± 0.10	0.78 ± 0.14	0.27
Cort. Por (%)	0.26 ± 0.03	0.23 ± 0.02	0.48	0.56 ± 0.12	0.73 ± 0.14	0.37
pMOI (mm^4^)	0.26 ± 0.01	0.35 ± 0.02	***0.002***	0.44 ± 0.02	0.49 ± 0.04	0.28

Cortical area, as assessed by μCT analysis of the midshaft femur, was significantly increased at 4 months in Dmp1-PPR^KO^ mice (Figure 1G, 1J, Table 2). Similarly, other cortical parameters, including polar moment of inertia, were also significantly increased in the mutant mice at 4 months of age, compared to controls whereas they were indistinguishable between the two genotypes at 13 months, indicating a differential temporal regulation of trabecular and cortical bone by PPR signaling in mature osteoblasts/osteocytes with age.

### PPR deletion in mature osteoblasts/osteocytes increases osteoclast, but decreases osteoblast, activity in 13-month-old mice

**Figure 2:**
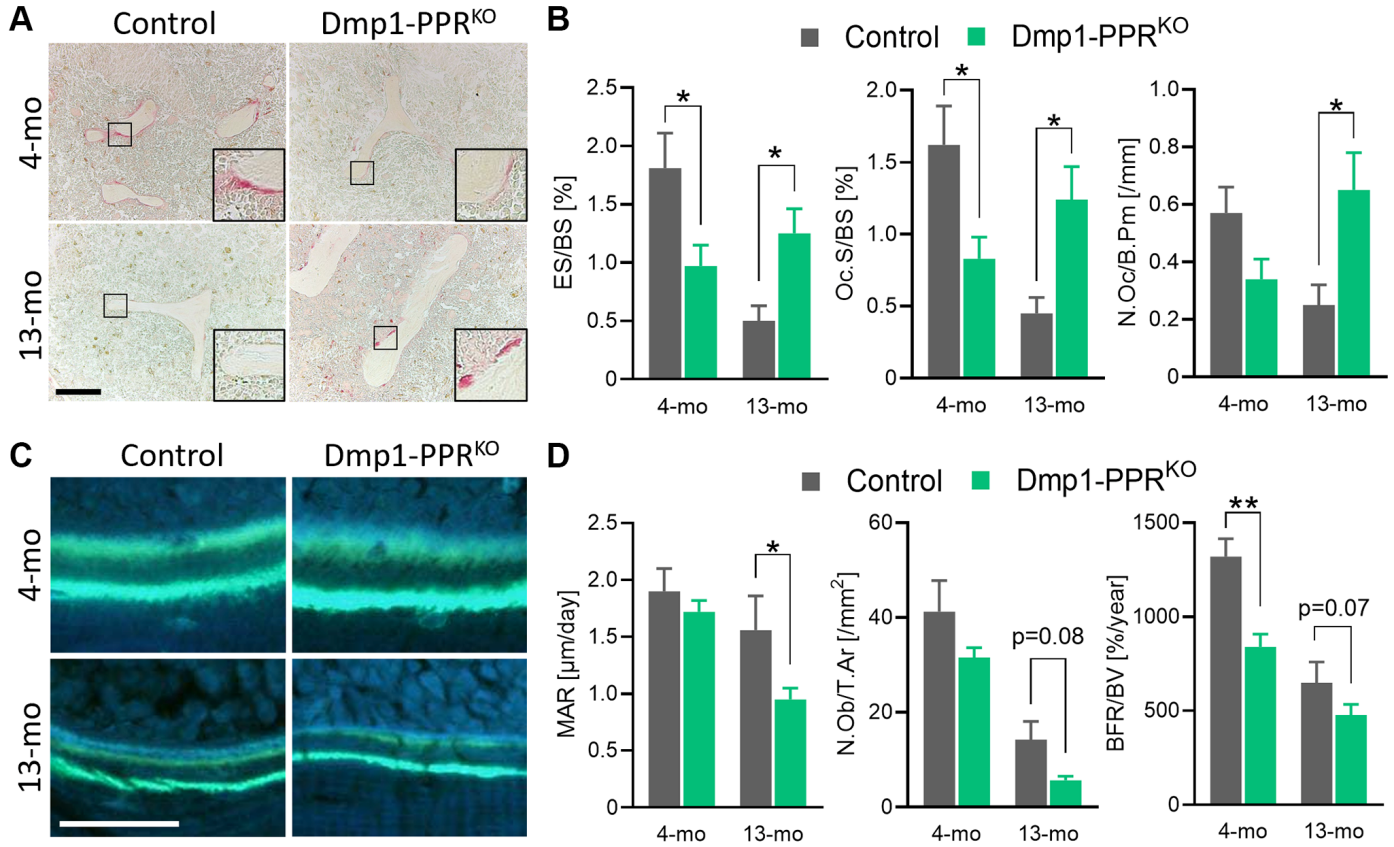
**Histomorphometric analysis of trabecular bones of Dmp1-PPR^KO^ mice.** (**A**, **B**) Representative TRAP staining images and bone resorption parameters of the distal femora from male control and KO animals. The inset shows a closeup displaying the TRAP-positive osteoclasts on the trabecular bone surface. Bar = 200 μm. (**C**) Representative images of calcein double-staining on the distal femora of these mice. Bone formation within 7 days was visualized by double calcein labeling. Bar = 50 μm. (**D**) Representative bone-formation parameters of the distal femora are shown. See Table 1 for the full list of resorption and formation parameters. *N* = 6–10 per group. Analyses were performed in a blinded fashion. Unpaired Student’s *t* test was performed. ^*^p < 0.05, ^**^p < 0.01. Data are presented as mean ± SEM. Abbreviations: ES: Erosion surface; BS: bone surface; Oc.S: osteoclast surface; N.Oc: number of osteoclasts; B.Pm: bone perimeter; MAR: mineral apposition rate; N.Ob: number of osteoblasts; T.Ar: tissue area; BFR: bone formation rate; BV: bone volume.

To delineate the cellular mechanism of the age-related osteopenia in the Dmp1-PPR^KO^ animals, we performed histomorphometric analysis on the L5 and the femora of adult and middle-aged mice. As shown in Figure 1C–1E, histomorphometric analysis confirmed the decrease in trabecular BV/TV% and Tb.N in male Dmp1-PPR^KO^ mice at 13 months, both in axial and appendicular sites. Tartrate resistant acid phosphatase (TRAP) staining on the distal femora of these mice showed relatively fewer osteoclasts per bone perimeter at 4 months, but strikingly more TRAP-positive cells at 13 months in Dmp1-PPR^KO^ animals as compared with controls (Figure 2A–2B). The significant increase in the number of TRAP-positive osteoclasts was also present in the proximal tibiae of 20-month-old Dmp1-PPR^KO^ female mice compared with control littermates (Supplementary Figure 2A). These findings were further supported by a significant reduction in osteoclast activity, such as erosion and osteoclast surface per bone surface, in 4-month-old Dmp1-PPR^KO^ mice but a significant increase of all these parameters at 13 months of age (Figure 2B, Table 1). Bone formation rate (BFR) over bone volume (BFR/BV) was significantly decreased in Dmp1-PPR^KO^ at 4 months and mineral apposition rate (MAR) was significantly reduced in 13-month-old Dmp1-PPR^KO^ mice compared to controls (Figure 2C–2D, Table 1). These results indicate that, in the absence of PPR signaling in mature osteoblasts/osteocytes, there is an age-dependent increase in osteoclast numbers and activity and age-independent decrease in osteoblast activity resulting in increased bone resorption and bone loss.

### Age-dependent changes in serum markers and skeletal genes in Dmp1-PPR^KO^ mice

**Figure 3:**
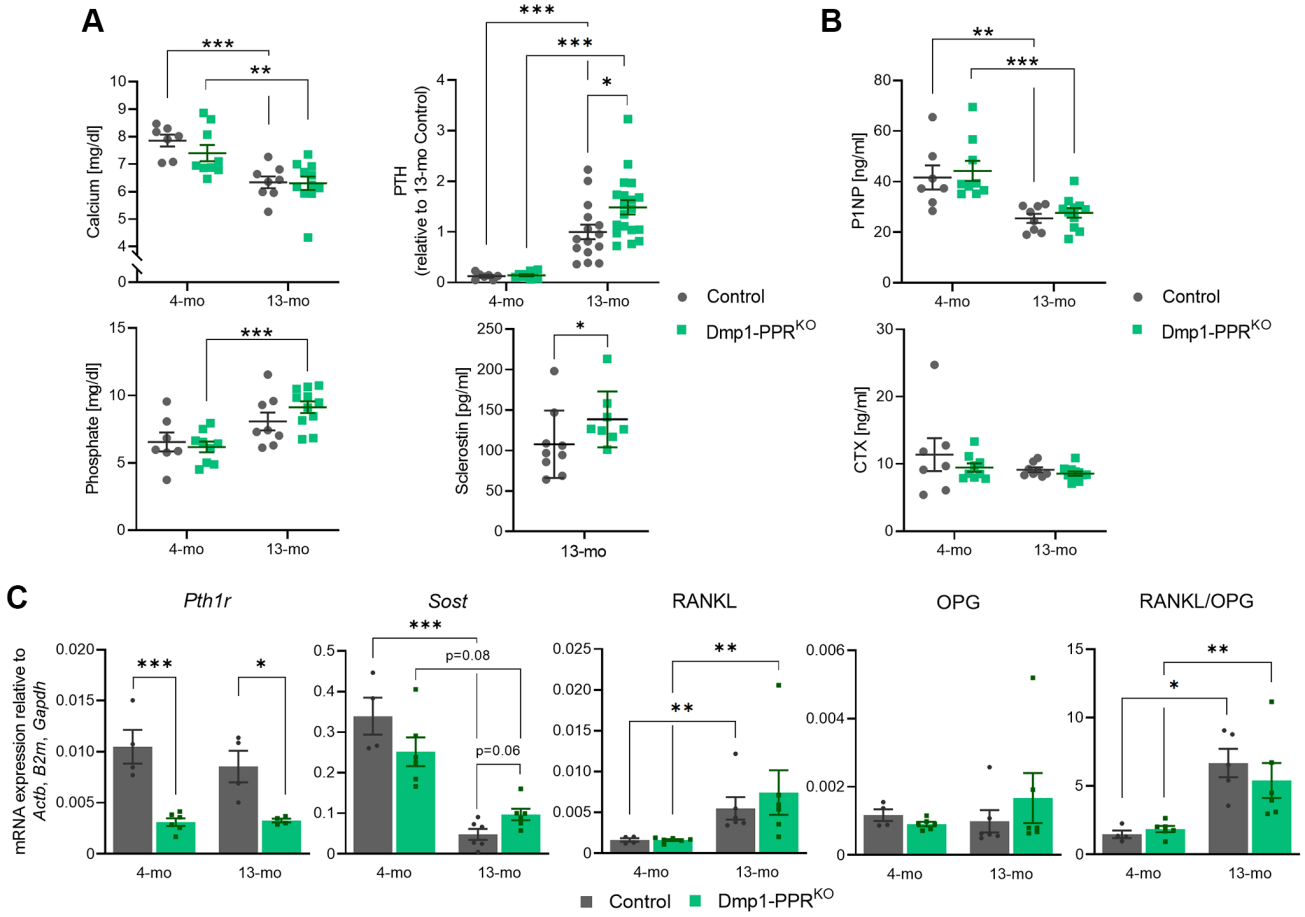
**Serum markers and skeletal gene expression in Dmp1-PPR^KO^ mice.** (**A**) Serum calcium, phosphate, PTH and sclerostin in male control and Dmp1-PPR^KO^ mice was measured by ELISA. PTH levels were normalized to 13-month-old control due to a high intra-assay variation (>9.8%). (**B**) Serum levels of bone formation (P1NP) and resorption marker (CTX) were also measured by ELISA. *N* = 7–19 per group. Data are presented as mean ± SEM. (**C**) Gene expression in marrow-removed long bones of male mice was analyzed with qPCR. *N* = 4–6 per group. Two-way ANOVA with Tukey’s *post hoc* test, unpaired Welch’s *t* test, or Mann-Whitney test was performed. ^*^p < 0.05, ^**^p < 0.01, ^***^p < 0.001. Each gene expression was relative to 3 housekeeping genes (*Actb*, *B2m*, and *Gapdh*), which were selected by GeNorm. Expression data are presented as mean ± SEM.

The main function of PTH is to maintain mineral homeostasis and it is still unclear whether mature osteoblasts/osteocytes directly contribute to mineral-ion homeostasis. To investigate if lack of PPR signaling in mature osteoblasts/osteocytes impaired mineral ions homeostasis, we measured serum levels of calcium, phosphate and PTH in adult and middle-aged mice. Biochemical analysis of male Dmp1-PPR^KO^ and littermate control mice showed normal calcemia and phosphatemia at 4 and 13 months of age (Figure 3A), demonstrating that PPR in mature osteoblasts/osteocytes is not required to maintain mineral-ion homeostasis. In both control and Dmp1-PPR^KO^, with age, there was a significant decrease in serum calcium and an increase in serum PTH, similar to what has been observed in older adult mice. PTH levels were similar between Dmp1-PPR^KO^ and controls at 4 months of age (Figure 3A), whereas they were significantly increased in Dmp1-PPR^KO^ mice at 13 months of age (Figure 3A), indicating a possible resistance to PTH. This increase in serum PTH in Dmp1-PPR^KO^ was not observed in 13-month-old female mice (Supplementary Figure 3B), suggesting the sex-dependent difference. Interestingly, phosphate serum levels significantly increased with age in Dmp1-PPR^KO^ mice but not in controls (Figure 3A). Serum markers of bone formation, procollagen type 1 N-terminal propeptide (P1NP), were significantly reduced in both male Dmp1-PPR^KO^ and controls at 13 months whereas markers of bone resorption, C-terminal telopeptide of type I collagen (CTX), were unchanged in both mice groups at both ages (Figure 3B), despite the significant increase in osteoclast numbers and activities present in Dmp1-PPR^KO^ mice at 13 months of age, as shown in Table 1 and Figure 2B.

To investigate the molecular mechanism leading to the increased osteoclasts in 13-month-old Dmp1-PPR^KO^ mice, we assessed the expression of PPR (encoded by *Pth1r*) and other osteocytic markers in bone marrow-deprived long bones of adult and middle-aged Dmp1-PPR^KO^ and control mice. Receptor ablation in osteocytes was still present in both male and female 13-month-old KO animals, as demonstrated by a significant decrease in PPR expression (Figure 3C, Supplementary Figure 4A). Although middle-aged male Dmp1-PPR^KO^ mice showed a trend of increase (2.0-fold, *p* = 0.06) in *Sost* expression compared to controls, overall *Sost* expression was unaffected by genotypes (Figure 3C). Interestingly, serum sclerostin was significantly increased in the mutant mice as compared to controls, which can contribute to the suppression of bone formation present in these animals (Figure 3A). Other osteocytic genes, namely RANKL and osteoprotegerin (OPG), were unchanged, suggesting that other factors might be driving the age-dependent increase in osteoclast numbers and activity present in middle-aged male Dmp1-PPR^KO^ mice. In the female mice, OPG expression was significantly downregulated, while the RANKL/OPG ratio was significantly increased, in KO mice at 13 months of age (Supplementary Figure 4A), demonstrating sex-dependent differences.

**Figure 4:**
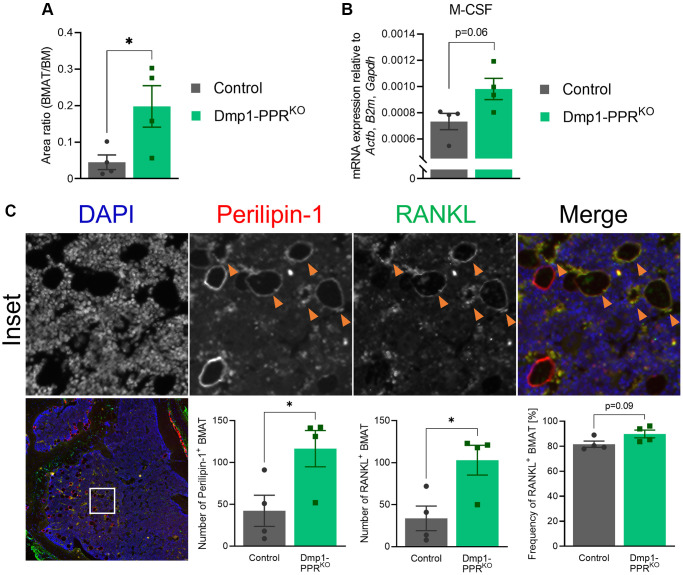
**Increased bone marrow adipocytes in middle-aged Dmp1-PPR^KO^ mice.** (**A**) The area of bone marrow adipose tissue (BMAT) over the total bone marrow (BM) space within 300-μm from the epiphyseal plate was analyzed on H&E-stained tibiae sections of male control and Dmp1-PPR^KO^ mice at 13 months of age. Representative images are shown in Figure 1A. *N* = 4 per group. (**B**) Expression of M-CSF in the BM isolated from the femora of middle-aged male animals (13 months old) was analyzed by qPCR. *N* = 4 per group. (**C**) Immunofluorescence staining of perilipin-1, RANKL and DAPI was performed on the tibiae of middle-aged (13 months) male control and Dmp1-PPR^KO^ mice. Representative images of a tibia from Dmp1-PPR^KO^ mouse are shown. In the merged image, DAPI, perilipin-1 and RANKL staining is shown in blue, red, and green, respectively. The orange arrowheads indicate RANKL^+^ BMAT (identified as perilipin-1^+^). The number of BMAT (left) and the number (middle) and frequency (right) of RANKL^+^ BMAT in the BM space were analyzed. *N* = 4 per group. Unpaired student’s *t* test was performed. ^*^p < 0.05. Data are presented as mean ± SEM.

Tumor necrosis factor α (TNFα) promotes osteoclastogenesis independently of RANKL [23]; therefore, we performed immunofluorescence staining for TNFα on the tibias of 13-month-old male mice and control littermates. In Dmp1-PPR^KO^ mice, the number of TNFα-expressing osteocytes was significantly decreased as compared to control littermates (Supplementary Figure 5), suggesting that other factors might drive osteoclastogenesis. In addition, we found a trend of increase (1.3-fold, *p* = 0.06) in M-CSF expression in the bone marrow of Dmp1-PPR^KO^ mice at 13 months of age compared to control littermates Figure 4B), and this upregulation of M-CSF in KO mice was also observed in 16-month-old mice (1.5-fold, *p* < 0.001, data not shown). Sphingosine kinase (Sphk1) mediates TNFα-induced arthritis and osteoclastogenesis via TNFα receptor activating factor 2 (TRAF2) [24]. We analyzed the expression of Sphk1 in the bone marrow of Dmp1-PPR^KO^ mice and found that Sphk1 was significantly upregulated as compared to control littermates (Supplementary Figure 6B), suggesting a potential involvement of TNFα-expressing osteocytes in the increase in M-CSF and Sphk1 expression in the bone marrow of Dmp1-PPR^KO^ mice. Next we measured serum levels of cytokines, previously reported to be regulated by PTH or by aging, including TNFα, monocyte chemoattractant protein 1 (MCP-1/CCL2), and interleukin (IL)-6 and -10 in serum of 13-month-old male mice (Supplementary Figure 3A). While there was a trend of increase (2.2-fold, *p* = 0.06) in serum IL-10 in Dmp1-PPR^KO^ compared to controls, serum concentration of other cytokines was unchanged.

### Serum from middle-aged Dmp1-PPR^KO^ male animals increases the number of osteoclasts *in vitro*

**Figure 5:**
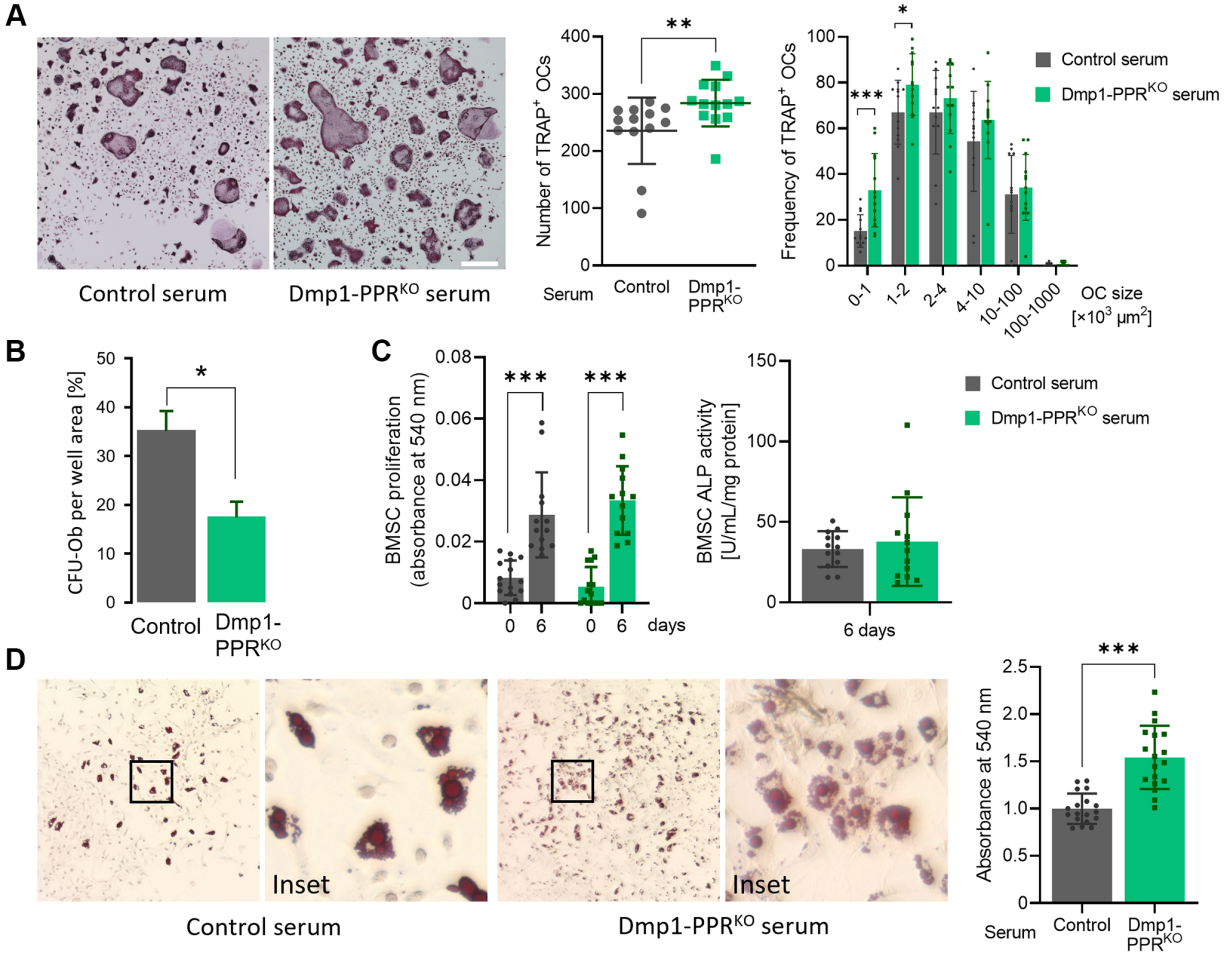
**Serum from Dmp1-PPR^KO^ mice increases osteoclastogenesis and adipogenesis.** (**A**) Representative TRAP staining images of BMMCs isolated from 3-month-old male control mice under osteoclastic differentiation in the presence of serum from 13-month old male control and KO mice. The total number (middle) and size distribution (right) of TRAP^+^ osteoclasts (OCs) per field were quantified. N = 13 per group. Data are presented as mean ± SEM. (**B**) CFU assay for osteoblasts was performed on BMSCs isolated from 13-month-old male control and Dmp1-PPR^KO^ animals. Data are presented as mean ± SEM. (**C**) Proliferation assay (left) and ALP activity assay (right) were performed on BMSCs isolated from 3-month-old male control mice under osteogenic differentiation in the presence of serum from male control and Dmp1-PPR^KO^ mice at 13 months of age. ALP activity assay was performed on day 6 of the osteogenic differentiation. *N* = 13–15 per group. (**D**) Representative Oil-red-O staining images of BMSCs isolated from 3-month-old male control mice under adipogenic differentiation in the presence of serum from male control and KO mice at 13 months of age. Quantification of lipid was performed by elution of Oil-red-O stain. *N* = 18 per group. Mann-Whitney test (**A**) or paired or unpaired *t* test (**B**–**D**) was performed. ^*^p < 0.05, ^**^p < 0.01, ^***^p < 0.001. Data are presented as mean ± SEM.

To explore the molecular mechanism(s) driving the osteopenia and the increase in osteoclast numbers and activity in middle-aged male Dmp1-PPR^KO^ mice, we treated bone marrow mononuclear cells (BMMCs) isolated from wild type mice (3–4 months old) with serum obtained from 13-month-old male mice (Dmp1-PPR^KO^ and littermate controls) and analyzed osteoclastogenesis by TRAP staining and activity. BMMCs treated with serum from KO mice significantly increased the number of TRAP^+^ osteoclasts compared to the control group (Figure 5A), whereas TRAP activity was similar between the two groups (Supplementary Figure 3C). These findings suggest that, in the absence of PTH signaling, osteocytes secrete factors that contribute, in part, to the increase in osteoclasts present in middle-aged Dmp1-PPR^KO^ mice.

### Osteoprogenitors are decreased in middle-aged Dmp1-PPR^KO^ animals

Next we assessed if lack of PPR signaling in mature osteoblasts/osteocytes influenced the commitment or frequency of osteoprogenitor cells, and therefore their osteogenic potential. As shown in Figure 5B, colony forming unit osteoblasts (CFU-Ob) were significantly reduced in bone marrow of male Dmp1-PPR^KO^ animals compared to controls at 13 months, demonstrating a progressive reduction in osteoprogenitors. Similarly, middle-aged female KO animals showed a marked reduction in CFU-Ob in the bone marrow (Supplementary Figure 3D).

### Serum from middle-aged Dmp1-PPR^KO^ male animals promotes adipogenic differentiation of BMSCs *in vitro*

Since circulating factors, including bone morphogenic proteins, are involved in osteolineage commitment of bone marrow stromal cells (BMSCs) [25], we examined the effect of serum from middle-aged mice on osteogenic differentiation of BMSCs. We treated BMSCs isolated from wild type mice (3–4 months old) with serum from 13-month-old male Dmp1-PPR^KO^ mice and littermate controls. Treatment with serum from control and KO mice showed no difference in proliferation and alkaline phosphatase (ALP) activity of BMSCs (Figure 5C), while BMSCs treated with serum from KO mice significantly increased adipogenic differentiation, as assessed by oil-red-O staining (Figure 5D). These findings demonstrate that circulating factors promote lineage commitment of BMSCs. To investigate if the secreted factors are osteocyte-derived, we treated BMSCs with conditioned medium from *ex vivo* culture of osteocyte-enrichment bone explants (OEBEs) from 13-month-old control and Dmp1-PPR^KO^ mice. Similarly, there was no difference in both BMSC proliferation and osteogenic differentiation between both treatment groups (Supplementary Figure 3E). These results indicate that factors secreted from osteocytes are not the major contributor to the reduced osteoprogenitors in Dmp1-PPR^KO^ mice.

### Marrow adipocytes are the source of RANKL

As demonstrated in Figure 5D, treatment with serum from 13-month-old Dmp1-PPR^KO^ mice promoted adipogenic differentiation of BMSCs *in vitro*. To examine if there was any change in marrow adiposity in these mice, we performed histological analysis on bone marrow. In Dmp1-PPR^KO^ male animals, at 13 months of age, there was a significant increase in marrow adiposity (Figures 1A, 4A). Since marrow adipocytes have been reported as a source of RANKL [26, 27], we analyzed RANKL expression in marrow adipocytes by immunofluorescence staining. Staining of RANKL along with an adipocyte marker, perilipin-1, revealed that the number of RANKL^+^ marrow adipocytes was markedly increased in Dmp1-PPR^KO^ compared to control male mice (Figure 4C). However, the ratio of RANKL^+^ adipocytes over total adipocytes was unchanged between control and mutant animals, suggesting that the increase in the number, but not the frequency, of RANKL^+^ adipocytes contributed to the increased osteoclast numbers in KO mice. It has also been reported that bone marrow adipocytes express M-CSF [28]. Interestingly, we found that M-CSF expression was upregulated (1.3-fold, *p* = 0.06) in the bone marrow of male Dmp1-PPR^KO^ mice as compared to controls (Figure 4B). This may indicate that the increased marrow adiposity also contributed to the upregulation of M-CSF in the middle-aged mutant mice. Expression of RANKL was unchanged in the bone marrow of 13-month-old male mice (Supplementary Figure 6A). Interestingly, the marrow adiposity was unchanged between female control and Dmp1-PPR^KO^ at 20 months of age, suggesting sexual dimorphism (Supplementary Figure 2B).

### PPR signaling protects osteocytes from early onset of oxidative stress *in vivo*

**Figure 6:**
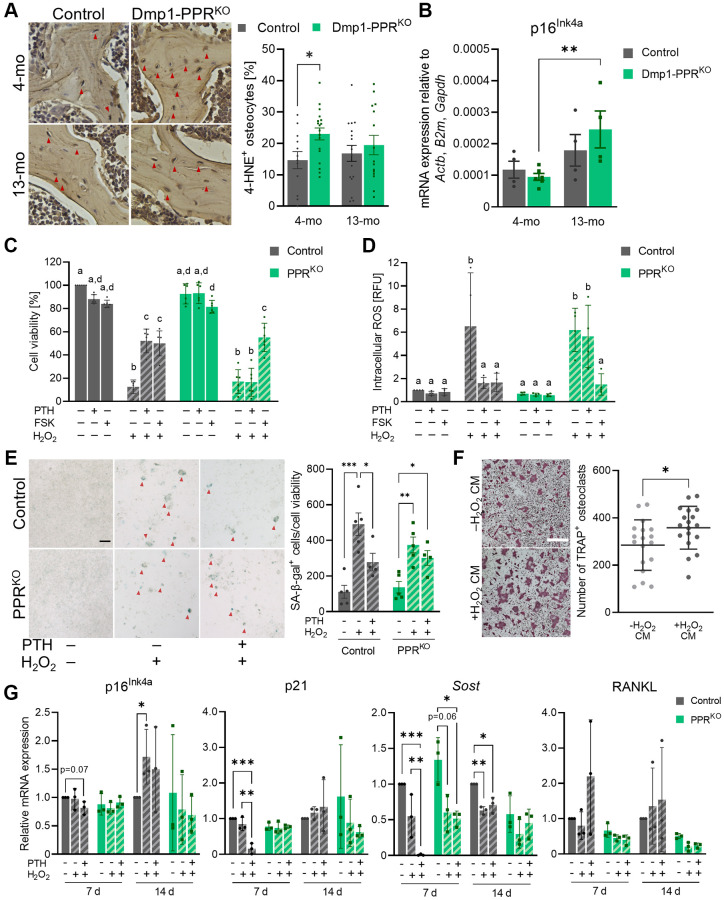
**PTH protects osteocytes from oxidative stress-induced cell death and senescence.** (**A**) Representative images of immunohistochemistry for 4-HNE on the L3/4 vertebrae from male animals are shown. The frequency of 4-HNE^+^ osteocytes per image field was analyzed. Mean ± SEM is shown. (**B**) Expression of p16^Ink4a^ in the tibiae of male control and Dmp1-PPR^KO^ mice was analyzed by qPCR. *N* = 4–6 per group. Mean ± SEM is shown. (**C**–**E**) Control and 12H-PPR^KO^ osteocytic cell line was pretreated with either 10 nM hPTH(1–34) or 10 μM forskolin (FSK) for 18-22 hrs prior to H_2_O_2_ exposure. (**C**) After H_2_O_2_ exposure (1 mM, overnight), cell viability was measured by resazurin-based assays. (**D**) After H_2_O_2_ exposure (1 mM, 4 h) intracellular ROS levels were measured using a fluorescent probe (DCFDA). Data are presented as relative fluorescence unit (RFU). (**E**) After continuous exposure to H_2_O_2_ (150 μM, 14 d), cells were stained for SA β-gal. Representative SA β-gal staining images and the quantification of SA β-gal^+^ cells (red arrowheads) are shown. Bar = 100 μm. (**F**) Representative TRAP staining images are shown of BMMCs isolated from 3–4-month-old male control mice under osteoclastic differentiation in the presence of conditioned medium from H_2_O_2_-treated control osteocytic cell line (–H_2_O_2_ or +H_2_O_2_, 150 μM for 7 days). Bar = 400 μm. *N* = 18 per group. (**G**) After continuous exposure to H_2_O_2_ (100 or 150 μM, 7 or 14 d), cells were harvested for RNA isolation. mRNA expression of p16^Ink4a^, p21, *Sost*, and RANKL were analyzed by qPCR. *N* = 3 per group. Kruskal-Wallis test with Dunn’s *post hoc* test, two-way ANOVA with Tukey’s *post hoc* test, one-way ANOVA with Sidak’s *post hoc* test or Mann-Whitney test were performed. ^*^p < 0.05, ^**^p < 0.01, ^***^p < 0.001. Same letter indicates n.s. Data are presented as mean ± SD.

Aging is accompanied by an accumulation of oxidative stress due to an imbalance between pro-oxidants and antioxidants [29, 30]. PTH protects osteoblasts from oxidative stress-induced cell death [31]; therefore we hypothesize that a similar effect was also present in osteocytes. To test the hypothesis, we analyzed the expression of 4-hydroxynonenal (4-HNE), a biomarker for oxidative stress-induced lipid peroxidation, in the L3/4 vertebrae of 4- and 13-month-old Dmp1-PPR^KO^ and control littermates. As shown in Figure 6A, Dmp1-PPR^KO^ mice exhibited a significant increase in 4-HNE-positive osteocytes at 4 months of age, suggesting the protective effect of PPR signaling in osteocytes from early onset of oxidative stress.

Oxidative stress is one of the causes of cellular senescence and it has been previously shown that senescent osteocytes contribute to age-related bone loss [32]. We investigated if the age-dependent bone loss in Dmp1-PPR^KO^ mice was driven by senescent osteocytes. The expression of the senescence marker p16^Ink4a^ was significantly increased in Dmp1-PPR^KO^ mice at 13 months of age compared to 4 months, whereas it was unchanged in controls (Figure 6B). Other markers of cellular senescence were unchanged (Supplementary Figure 4C).

### PTH protects osteocytes from oxidative stress-induced cell death and intracellular ROS accumulation *in vitro*

To study the effects of PTH in oxidative stress, we used an *in vitro* model in which PPR expression was knocked-out by CRISPR/Cas9 technique. In the absence of PPR expression in Ocy454-12H cells [33] (12H PPR^KO^) (Supplementary Figure 7A–7C), there was a significant decrease in the basal RANKL and RANKL/OPG expression (Supplementary Figure 7C), which is similar to the phenotype present in 3-month-old Dmp1-PPR^KO^ animals [22], whereas *Sost* expression was unchanged compared to control cells. 12H-PPR^KO^ cells were treated with hPTH(1–34) or forskolin for 18–22 hrs and then exposed to a high dose of H_2_O_2_ (1 mM). As shown in Figure 6C–6D, PTH treatment significantly suppressed oxidative stress-induced cell death and intracellular accumulation of ROS in control cells, whereas this effect was lost in 12H-PPR^KO^ cells, demonstrating that PTH protects osteocytes from oxidative stress-induced cell death.

### PTH protects osteocytes from oxidative stress

ROS accumulation promotes oxidative stress and cellular senescence, therefore we evaluated the effects of PTH during oxidative stress. Control and 12H-PPR^KO^ cells were pretreated with hPTH(1–34) prior to 7 and 14 days of continuous exposure to a low dose of H_2_O_2_ (150 μM). In the non-pretreated groups, H_2_O_2_ exposure markedly increased the number of senescence-associated β-galactosidase-positive (SA β-gal^+^) cells in both control and 12H-PPR^KO^ cells, while PTH-pretreatment significantly reduced the number of SA β-gal^+^ cells in control, but not in 12H-PPR^KO^ cells (Figure 6E, Supplementary Figure 8A). Gene expression in these cells after 7 and 14 days of H_2_O_2_ (100–200 μM) exposure (Figure 6G) demonstrated that PTH had a long-lasting effect on *Sost* and RANKL expression. Importantly, pretreatment of control, but not 12H-PPR^KO^, cells with PTH significantly suppressed p21 expression at day 7, demonstrating a potential molecular mechanism by which PTH protects osteocytes from oxidative stress-induced senescence. Expression of p16^Ink4a^ was significantly upregulated in control cells exposed to H_2_O_2_ alone (–PTH) at day 14 (Figure 6G).

Next we assessed whether factors secreted by osteocytes under oxidative stress promote osteoclastogenesis. Conditioned medium (CM) from cells treated with H_2_O_2_ (+H_2_O_2_ CM, 7 days) (both without PTH pretreatment) was used to treat BMMCs. The BMMCs treated with +H_2_O_2_ CM increased osteoclast numbers compared to CM control (–H_2_O_2_ CM) (Figure 6F), demonstrating that, under oxidative stress, osteocytes secrete osteoclastogenic factors, which might contribute to the increased osteoclasts in Dmp1-PPR^KO^ mice. Treatment with H_2_O_2_ medium alone did not increase osteoclast numbers compared to CM control (data not shown).

Interestingly, when 12H-PPR^KO^ cells were pre-treated with PTH and then exposed to γ-irradiation (5 Gy) or busulfan (50 μM, 7 days), an alkylating agent [34], PTH had no effect (Supplementary Figure 8B).

Taken together these results demonstrate that, in the absence of PPR signaling in mature osteoblasts/osteocytes, there is an age-dependent trabecular bone loss associated with increased bone resorption driven by a significant increase in osteoclast numbers and activity and impaired bone formation. Mechanistically, PPR signaling in mature osteoblasts/osteocytes regulates osteoblast formation through serum sclerostin and osteoclastogenesis via secreted factors other than RANKL and OPG. At the molecular level, PPR signaling protects, *in vitro*, osteocytes from oxidative stress-induced cell death through a cAMP-mediated mechanism. Further studies will be needed to elucidate the downstream effectors.

## DISCUSSION

Over the last two decades, the actions of PTH have expanded to include important effects on skeletal homeostasis and hematopoiesis. In particular, the amino-terminal fragment of PTH and PTHrP were approved by FDA as therapeutic agents capable of restoring bone mass and increasing the number of hematopoietic stem cells [1, 35, 36]. It has been shown that the anabolic effects of PTH in bone comprise the recruitment of osteoblast progenitors, the suppression of osteoblasts and osteocytes apoptosis, the suppression of *Sost*/sclerostin expression and the activation of bone lining cells. Although the cellular targets of these actions are still not completely understood, the use of genetically manipulated animals has shed light on some of the hormonal actions. Using transgenic mice in which the PTH receptor is ablated predominantly in mature osteoblasts and osteocytes, we have demonstrated that receptor expression in these cells was required for bone modeling and remodeling and for a full anabolic and catabolic response to PTH administration [22].

**Figure 7:**
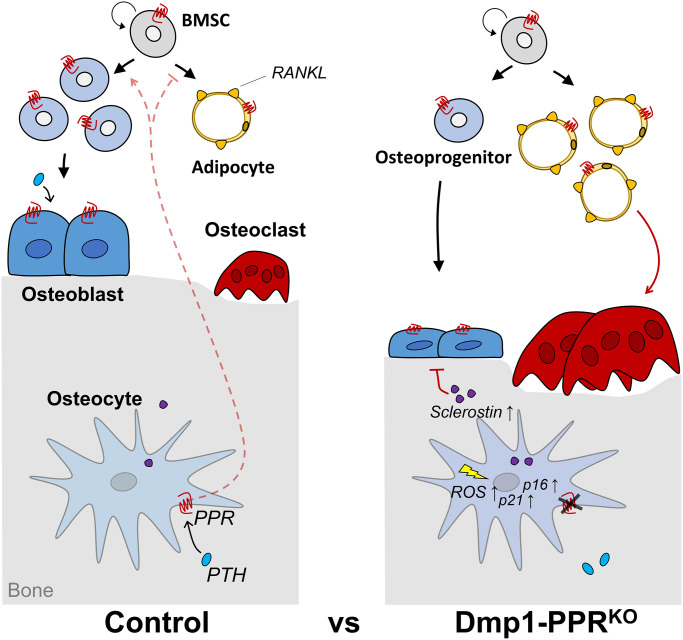
**Graphical summary.** 13-month-old Dmp1-PPR^KO^ mice showed trabecular bone loss driven by increased osteoclast number and activity and reduced osteoblast function. Mechanistically, the lack of PPR signaling in mature osteoblasts/osteocytes decreases osteoprogenitors and increases serum sclerostin, RANKL-expressing marrow adipocytes and early onset of 4-HNE+ osteocytes and p16^Ink4a^ upregulation in KO mice. Circulating factor(s) from these mutant mice increases, directly or indirectly, osteoclastogenic and adipogenic differentiation of BMMCs and BMSCs, respectively. Furthermore, *in vitro* data showed that PPR signaling induces long-lasting suppression of p21 and protects osteocytes from oxidative stress-induced intracellular ROS accumulation, cell death and senescence.

Here we report that, with aging, Dmp1-PPR^KO^ mice develop a significant osteopenia characterized by reduced trabecular bone, whereas the cortical compartment is relatively unaffected. The bone loss is driven by an increase in the number of osteoclasts, their surface area and activity and a concomitant reduction in osteoblast activity (Figure 7). In contrast, at 4 months of age, and as previously reported for 3-month-old animals [22], Dmp1-PPR^KO^ mice have increased trabecular bone associated with reduced osteoclast and osteoblast functions. These findings demonstrate that PPR signaling in osteocytes exerts different effects on trabecular and cortical bone and that these effects are age dependent and shift, with age, from maintenance of bone homeostasis to prevention of bone loss. Interestingly, recent transcriptomic profiles of skeletal tissue in male and female mice over their lifespan identified *Pth1r* as one of the genes highly dependent on both gender and age [37]. We identify a notably sex-dependent difference in our study (8.67-fold-increase of RANKL in male vs. female at 13-month-old, *p* < 0.001) that can be explained in light on the *Pth1r* differential expression (1.72-fold-increase in male vs. female at 13-month-old, *p* = 0.07).

Considering the classical role of PTH in calcium homeostasis, we investigated whether PPR signaling in mature osteoblasts/osteocytes is required in maintaining mineral ion homeostasis. There was a significant age-related decrease in serum calcium and increase in serum PTH in both genotypes. Notably, 13-month-old Dmp1-PPR^KO^ male mice had a higher serum PTH level than littermate controls, whereas no difference was found between female Dmp1-PPR^KO^ mice and controls. The significant increase in serum PTH in Dmp1-PPR^KO^ males could be due to an age-dependent resistance to PTH. Elevated serum PTH levels might be indicative of a secondary hyperparathyroidism-like state, which may contribute to the accelerated bone loss in Dmp1-PPR^KO^ mice. Despite the elevated serum PTH, osteoblasts were not increased, most likely due to the concomitant increase in serum sclerostin. In addition, in the Dmp1-PPR^KO^ mice, there is an age-dependent exhaustion of osteoprogenitor cells, as supported by a reduction in CFU-Ob. Phosphate levels were also increased in male Dmp1-PPR^KO^ mice with age, whereas were unchanged in the control mice. Since PTH increases FGF23 in osteocytes [38], Dmp1-PPR^KO^ mice might have a lower FGF23 secretion than control littermates, which can lead to reduced phosphate excretion in the kidneys.

To delineate the molecular mechanisms by which Dmp1-PPR^KO^ animals display severe bone loss with age, we analyzed the expression of osteocytic markers. Despite the important role of osteocytes in regulation of both osteoblasts and osteoclasts, expression of *Sost*, RANKL, and OPG was similar between middle-aged male Dmp1-PPR^KO^ and littermate controls. Contrarily, in 13-month-old Dmp1-PPR^KO^ females, OPG and RANKL/OPG expression were significantly suppressed and increased, respectively, as compared to littermate controls. In male mice, *Sost* and RANKL expression at 4 months of age was unchanged between genotypes, whereas we previously reported an increase and decrease in *Sost* and RANKL expression, respectively, in KO animals at 3 months of age [22]. At the moment we do not have any explanation for this age-dependent difference and further studies will be needed.

RANKL, OPG, and RANKL/OPG expression were unchanged in the long bones of male mice, suggesting that additional cytokines might be involved in the increase in osteoclast numbers and activity. Serum TNFα levels were unchanged between control and mutant mice at 13 months of age, and TNFα expression in osteocytes, as assessed by immunofluorescence, was decreased in long bones of mutant mice compared to controls, indicating that additional factors might be involved. Bone marrow adipocytes are responsive to PTH and are a local source of RANKL; therefore we investigated if increased osteoclast activity in Dmp1-PPR^KO^ mice was dependent on these cells. Indeed, we found that, with aging, there was a significant increase in RANKL+ adipocyte in the bone marrow of mutant mice, as compared to controls. Treatment of BMMCs with serum of Dmp1-PPR^KO^ mice induced a significant increase in osteoclasts as compared to controls, demonstrating the presence of osteoclastogenic factors in the serum of mutant mice. We previously identified osteocyte-derived molecules that modulate osteoclast numbers and activities, such as Nrp1, Sema3a, and Sema3d [39]. RNA-sequencing of osteocytes [40] demonstrated that PTH significantly increased the expression of *Nrp1* (2.52-fold increase, *p* < 0.001) in these cells, suggesting a possible mechanism of action.

Serum CTX is often used as a marker of bone resorption and, despite the severe bone loss and the significant increase in osteoclasts, we did not detect any significant changes in serum CTX in Dmp1-PPR^KO^ mice, compared to controls. It has been reported that systemic CTX levels vary according to circadian rhythm and that food intake reduces the levels of CTX in humans [41, 42]. Moreover, CTX measurements can be affected by lipids and proteins present in the serum after food intake. Since mice were not starved before serum collection, it is possible that the levels of CTX measured were not accurate.

PTH reduces the rate of both osteoblast and osteocyte apoptosis, which releases factors capable of promoting osteoclastogenesis [43–45]. We investigated whether the osteopenia present in the Dmp1-PPR^KO^ animals at 13 months old was due to increased osteocyte apoptosis. Terminal deoxynucleotidyl transferase deoxyuridine triphosphate nick-end labeling (TUNEL) assay showed no difference in osteocyte apoptosis in both controls and Dmp1-PPR^KO^ mice, both in males and females (Supplementary Figure 2C, 2D), suggesting that mechanisms other than osteocyte apoptosis are responsible for the osteopenia.

We demonstrated that 13-month-old Dmp1-PPR^KO^ mice have reduced CFU-Ob and increased marrow adiposity compared to controls, suggesting that the stem cell population might be depleted. Similarly, we observed the reduced expression of PPR in bone marrow cells in Dmp1-PPR^KO^ male mice, but not in females (Supplementary Figure 6A, 6C). Treatment of BMSCs with serum from Dmp1-PPR^KO^ mice induced adipogenic differentiation but had no effect on osteoblastic differentiation. It has been reported that ceruloplasmin (Cp), a multicopper ferroxidase (also known as an adipokine), increases adipogenic differentiation of MC3T3 cells [46]. Cp is also the one of the osteocyte-secreted proteins that we identified previously [39] and PTH has been reported to reduce mRNA expression of Cp in osteocytes [40], indicating the possible involvement of Cp in the increase in marrow adiposity in KO mice. We also observed a trend of increase in *Sost* expression in long bone of 13-month-old Dmp1-PPR^KO^ male mice compared to controls and a significant increase in serum sclerostin. We can speculate that sclerostin may contribute to the reduction in osteoblast proliferation, possibly in favor of marrow adipocytes, by suppressing the Wnt/β-catenin signaling pathway. Indeed, it has also been reported that sclerostin reduces proliferation and differentiation of BMSC, in part by suppressing bone morphogenetic protein activity and by increasing bone marrow adipose tissue (BMAT) [19, 47]. Further studies will be needed to identify the molecular mechanism(s) by which osteocytes affect the BMSCs.

Aging is characterized by reduced skeletal mass and increased oxidative stress and recent studies identified senescence and senescent osteocytes as important players in age-dependent bone loss [32, 48]. The aging process comprises not only cellular dysfunction and genomic instability but also stem cell exhaustion. Several studies have demonstrated that the decline in bone formation that occurs with age, both in humans and animal models, is invariably associated with reduced proliferation and differentiation of mesenchymal stem cells. Here we report that the absence of PPR expression in mature osteoblasts/osteocytes in mice induces a significant increase in the number of 4-HNE^+^ osteocytes in 4-month-old mutant mice compared to control littermates and a marked increase in p16^Ink4a^ expression at 13 months of age. These findings suggest that PTH protects osteocytes from early onset of oxidative-stress. Further studies are needed to unveil the underlying molecular mechanism.

Our *in vitro* study showed PTH protects osteocytes from oxidative stress-induced cell death and intracellular accumulation of ROS in a cyclic AMP-dependent manner. These results suggest that signaling through PPR, which is expressed on these cells and some mature osteoblasts, is needed to protect osteocytes from oxidative stress and possibly cellular senescence. One mechanism by which Dmp1-PPR^KO^ animals become severely osteopenic is by increased production of ROS, due to reduced activities of antioxidants during aging [29]. Indeed, the role of ROS and PTH in osteoblastic cells has been documented by the work of Jilka et al. [45], demonstrating that intermittent PTH administration reduces intracellular ROS and p66^shc^ phosphorylation.

Autophagy has also recently been shown to play an important role in aging and senescence. To investigate a possible relationship between PPR signaling in osteocytes and autophagy, we analyzed expression of transcripts known to regulate autophagy, such as Sirt-1, FOXO-1 and Beclin-1. However, we found no changes in expression of these genes in our mutant mice (Supplementary Figure 4B).

It has been previously reported that, similar to PTH, PTHrP protects osteoblastic cells from oxidative stress. Ardura et al. reported that PTHrP counteracts the pro-apoptotic actions of ROS by modulating mitogen-activated protein kinases (MAPK) phosphatase 1 (MAPK1) and promoting dephosphorylation of MAPK [49]. Mice with the “knock-in” (KI) of the 1-84 fragment of PTHrP, which lacks both the nuclear localization sequence (NLS) and the C-terminus, display early senescence and defective osteoblast functions. In these animals, ROS levels are increased and antioxidant enzymes are downregulated, demonstrating a role for PTHrP in prevention of oxidative stress [50–52]. Since PTH and PTHrP both bind to and activate the PPR, it is plausible to hypothesize that they might exert similar effects. Additional studies will be needed to analyze PTH and PTHrP responses. Interestingly, PTHrP mRNA expression in long bone of control and Dmp1-PPR^KO^ mice was similar, therefore we can speculate that this effect was not dependent on PTHrP.

In summary, we have reported, for the first time, that PPR signaling in mature osteoblasts/osteocytes is needed to protect the skeleton from age-dependent bone loss. In Dmp1-PPR^KO^ mice, there is a striking age-dependent trabecular bone loss driven by increased osteoclast number and activity and reduced osteoblast function. Mechanistically, we demonstrated that lack of PPR signaling decreases osteoprogenitors while increasing serum sclerostin, RANKL-expressing marrow adipocytes and 4-HNE^+^ osteocytes. Moreover, there was an age-dependent upregulation of p16^Ink4a^ in KO mice. We also found that circulating factor(s) from these mutant mice increases, directly or indirectly, osteoclastogenic and adipogenic differentiation of BMMCs and BMSCs, respectively. Furthermore, *in vitro* data showed that PPR signaling induces long-lasting suppression of *Sost* and p21 and protects osteocytes from oxidative stress-induced intracellular ROS accumulation, cell death and possibly senescence (Figure 7).

## MATERIALS AND METHODS

### Mice

Dmp1-PPR^KO^ (Dmp1-Cre^+^; PPR^fl/fl^) mice under the C57BL/6 genetic background were generated as described previously [22]. Littermates homozygous for floxed PPR gene, but lacking Cre-recombinase expression, were used as control (Dmp1-Cre^–^;PPR^fl/fl^). Details are provided in Supplementary Methods. Institutional Animal Care and Use Committee and the Subcommittee on Research Animal Care at Massachusetts General Hospital and Boston University Medical Center approved all animal protocols.

### Cell lines

Ocy454-12H (or 12H) cells, a derivative of the conditionally immortalized osteocytic cell line Ocy454, were used for all the *in vitro* experiments [33, 53, 54]. CRISPR/Cas9 genome editing technique was used to knockout PPR expression in these cells (12H-PPR^KO^). Both control and 12H-PPR^KO^ cells were routinely maintained at 33°C (permissive temperature) with 5% CO_2_ and cultured in growth medium (α minimum essential medium (Gibco, Thermo Fisher Scientific) containing 10% heat-inactivated fetal bovine serum (Gibco) and 1% antibiotic-antimycotic (Gibco)). Upon proliferation, cells were transferred to 37°C with 5% CO_2_ to be fully differentiated into mature osteocytes and incubated for the number of days required for each experiment. Fresh growth medium was added every 3–4 days. Details are provided in Supplementary Methods.

### Immunofluorescent staining

Immunofluorescent staining for RANKL and perilipin-1 was performed on paraffin sections of the left tibiae from middle-aged (13 months old) male mice. See Supplementary Methods for details. The following antibodies were used: anti-mouse perilipin-1 (#9349, Cell Signaling Technology, Danvers, MA, USA) (1:100), anti-mouse RANKL (#sc-7628, Santa Cruz Biotechnology, Dallas, TX, USA) (1:50), Alexa flour 546 donkey anti-rabbit IgG (#A10040, Invitrogen, Carlsbad, CA, USA) (1:100) and Alexa flour 488 donkey anti-goat IgG (#A11055, Invitrogen) (1:100).

### Senescence-associated β-gal staining

Cells were plated at a density of 1.0 × 10^5^ cells/ml in growth medium on a 6- or 12-well plate (Biolite™) under 37°C with 5% CO_2_ followed by treatment, on the next day, with 10 nM hPTH(1–34) for 18–22 hrs. Cells were then exposed to oxidative stress by adding an equal volume of 300 μM H_2_O_2_ medium (final concentration: 150 μM) on top of the well and cultured for an additional 7 or 14 days. Medium was replaced once on day 7. On day 7 and 14, conditioned medium was collected and the cells were either harvested for RNA isolation or fixed with 4% paraformaldehyde (Acros Organics, Fair Lawn, NJ, USA) in PBS for 15 min at room temperature for SA β-gal staining. Cells were then washed with PBS and incubated with SA β-gal staining solution (pH 6.0) overnight at 37°C without CO_2_ injection. The SA β-gal staining solution was prepared by following the protocol from Chen *et al.* [55]. Cell viability was measured using PrestoBlue cell viability reagent (Invitrogen) as per manufacturer’s instruction. Bright field images were acquired under the microscope with a 4X objective (Keyence). The number of SA β-gal positive osteocytes was counted in a blinded fashion using ImageJ.

### Oxidative stress-induced cell death and reactive oxygen species accumulation

Cells were plated at a density of 2.6 × 10^4^ cells/cm^2^ in growth medium on a 24-well plate and grown for 3 days at 33°C. The growth medium was replaced and the cells were cultured for an additional 2 days under 37°C and treated with either 10 nM hPTH(1–34) or 10 μM forskolin for 18–22 hrs followed by an exposure to 1 mM H_2_O_2_ for 4 hrs or overnight. Cell viability was measured using PrestoBlue cell viability reagent as per manufacturer’s instruction. Intracellular levels of ROS were determined upon cell staining with 14 μM of 2′, 7′-dichlorofluorescin diacetate (DCFDA) (Sigma) for 30 min at 37°C with 5% CO_2_. The cells were then trypsinized and transferred into wells of a 96-well plate and fluorescence (485 nm excitation and 535 nm emission) was measured using a spectrophotometer (TriStar LB941, Berthold Technologies, Oak Ridge, TN, USA). The fluorescent intensity was normalized with cell viability.

### Statistics

Normal distribution and heteroscedasticity of the data was tested using D'Agostino-Pearson or Anderson-Darling test and Spearman's test, respectively. Equality of variance between two datasets was analyzed with the F test. Parametrical statistics were used for the data that follow normal distribution and/or equal variance. Otherwise, non-parametrical statistics were used. Statistical significance was defined as *p* values less than 0.05 (two-tailed). Statistical test used for each analysis is described in the legends of each figure. All statistical analysis was performed on GraphPad Prism (GraphPad Software, San Diego, CA, USA).

## Supplementary Materials

Supplementary Methods

Supplementary Figures

Supplementary Tables
