# Supplementary material for: Parathyroid hormone signaling in mature osteoblasts/osteocytes protects mice from age-related bone loss
Source: Aging (Albany NY). 2021 Dec 30;13(24):25607–42. doi: 10.18632/aging.203808 (PMC8751595; doi:10.18632/aging.203808)
Supplement: Supplementary Methods [file aging-13-203808-s001.pdf]

SUPPLEMENTARY METHODS

Mice

Mice were maintained under standard laboratory conditions on a 12:12 hr light;dark cycle. Water and standard chow (#2918, Teklad global 15% protein, Envigo, Indianapolis, IN, USA, or #5V75, LabDiet PicoLab crude 20% protein, PMI Nutrition International, St. Louis, MO, USA) was provided ad libitum. Mice were housed in ventilated cages at 4–5 animals per cage. Male (4 and 13–16 months old) and female (13–20 months old) mice were used for this study.

All mice were euthanized by cervical dislocation following isoflurane (Henry Schein, Melville, NY, USA) anesthesia. Femora and tibiae from both sides and vertebrae were dissected and cleaned of soft tissue. Left femora were used for μCT analysis and subsequently used for either histomorphometric analysis (described below). Right femora were used for histological analysis (described below). The L5 vertebrae were used for μCT analysis and subsequently used for histomorphometric analysis. The third and fourth lumbar (L3 and L4) vertebrae were used for histological analysis (described below). The right femora and/or tibiae were used for RNA isolation and gene expression analysis (described below).

Cell lines

CRISPR/Cas9 genome editing technique was used to knockout PPR expression in Ocy454-12H (or 12H) cells. Briefly, three independent single-guided RNAs (sgRNAs) were designed using both sgRNA Design Tool (Zhang Lab, MIT, http://crispr.mit.edu/) and sgRNA Designer Ver.1 (Genetic Perturbation Platform Web Portal, Broad Institute). Each sgRNA was cloned into a pSpCas9(BB)-2A-GFP (PX458) plasmid (#48138) (a gift from Dr. Feng Zhang [1], Addgene, Watertown, MA, USA). The plasmid was then transfected into Ocy454-12H cells with FuGENE HD (Promega, Madison, WI, USA) following the manufacturer's protocol. Two days after transfection, enhanced green fluorescent protein expressing cells (12H-PPR^{KO}) were sorted using fluorescence-activated cell sorting technique (MoFlo Astrios, Beckman Coulter, Brea, CA, USA). Successful knockout of the PPR gene (*Pth1r*) and the loss of PPR signaling in 12H-PPR^{KO} cells was confirmed by quantitative real time polymerase chain reaction (PCR) (StepOne Plus, Applied Biosystems, Foster City, CA, USA) and direct cAMP radioimmunoassay (RIA) (MGH Center for Skeletal Research Cores, Boston, MA, USA), as described below, along with DNA sequencing (Eton Bioscience, Charlestown, MA, USA) (Supplementary Figure 7). An empty PX458 plasmid (without any sgRNA insertion) was used to generate control cells (12H-PPR^{Cont}). Both control and 12H-PPR^{KO} cells were routinely maintained at 33°C (permissive temperature) with 5% CO_2 and cultured in growth medium (α minimum essential medium (αMEM) (Gibco, Thermo Fisher Scientific) containing 10% heat-inactivated fetal bovine serum (FBS) (Gibco) and 1% antibiotic-antimycotic (Gibco)). Upon proliferation, cells were transferred to 37°C with 5% CO_2 to be fully differentiated into mature osteocytes and incubated for the number of days required for each experiment. Fresh growth medium was added every three-four days.

Human PTH 1-34 (hPTH(1–34)) peptide (MGH Peptide Core Facility) was dissolved in 0.1% trifluoroacetic acid at 1 mM, aliquoted, stored at –80°C, and subsequently diluted to the appropriate concentration in growth medium. For the relevant experiments, cells were treated with hPTH(1–34) or forskolin (Sigma-Aldrich, St. Louis, MO, USA) for 4 or 18–22 hrs and the total RNA was isolated using RNeasy Plus Mini Kit (Qiagen, Valencia, CA, USA).

Colony forming unit assay

Upon sacrifice, BMSCs were isolated from long bones (tibiae and/or femora) of 13-month-old control and Dmp1-PPR^{KO} animals (both male and female) by either a brief centrifugation or flushing out bones using a 25G needle (BD, Franklin Lakes, NJ, USA) with αMEM containing 0.1% bovine serum albumin (BSA) (Sigma) and 25 mM HEPES buffer (Fisher Scientific, Hampton, NH, USA). Cells were hemolyzed with red blood cell lysing buffer (Sigma) and plated on either 6- or 24-well plates (Biolite™, Thermo Fisher Scientific, or Primaria™, Corning, Corning, NY, USA) at 1.0–2.0 × 10^6 cells/well under growth medium. Cells were cultured at 37°C with 5% CO_2 for 2 days and non-adherent cells were removed by replacing the growth medium with osteogenic differentiation medium (growth medium supplemented with 10 mM β-glycerophosphate (Calbiochem, Millipore, Burlington, MA, USA), and 50 μg/ml of ascorbic acid (Sigma)). The differentiation medium was replaced every 2–3 days. After 6–12 days in culture, cells were fixed with 10% phosphate-buffered formalin (Fisher Scientific) and stained for alkaline phosphatase (ALP) by incubating with 0.1 mg/ml naphthol AS-MX phosphate (#N5000, Sigma) and 0.6 mg/ml Fast Blue BB salt (#151112, MP Biomedicals, Santa Ana, CA, USA) dissolved in 0.1 M Tris buffer (pH 8.5) (Fisher Scientific). CFU-Ob assay was performed by quantifying the number or the area of colonies that are

positive for ALP as previously reported [2, 3]. Cells were subsequently stained with 0.05% crystal violet (CV) for the fibroblasts CFU (CFU-F) assay and the total area of CV-positive colonies was quantified using ImageJ (NIH, Rockville, MD, USA).

Histology

The left tibiae and/or the L3 and L4 vertebrae were dissected from adult (4 months old) and middle-aged male (13–16 months old) or female (13–20 months old) mice and were fixed in 10% phosphate-buffered formalin (pH 6.9–7.2) for at least 24 and 48 hrs, respectively. The bones were further decalcified in 20% ethylenediaminetetraacetic acid (EDTA) (Fisher Scientific or Alfa Aesar, Haverhill, MA, USA), processed, embedded in paraffin, and sectioned as described previously (12). Following deparaffinization, sections were stained with hematoxylin (Thermo Fisher Scientific) and eosin (Fisher Scientific) or used for immunofluorescent staining, TUNEL assay, or TRAP staining (each described below).

Immunofluorescent staining

Immunofluorescent staining for TNFα, RANKL and perilipin-1 was performed on paraffin sections of the left tibiae from middle-aged (13 months old) male mice. Antigen was retrieved by Tris-EDTA buffer (10mM Tris, 1mM EDTA, 0.05% Tween 20, pH 9.0) in a boiling water bath at 95°C for 12 min for TNFα or overnight incubation at 60°C in sodium citrate buffer (10 mM sodium citrate (Fisher Scientific), 0.05% Tween 20 (Sigma), pH 6.0) for RANKL and perilipin-1. Slides were permeabilized with 0.1% Triton X-100 (Sigma) in phosphate buffered saline (PBS) at room temperature for 10 min (for RANKL and perilipin-1) and then blocked with TNB blocking buffer (20 mM Tris, 150 mM NaCl (Fisher Scientific), 0.5% tyramide signal amplification (TSA) blocking reagent (#FP1020, PerkinElmer, Waltham, MA, USA)) for 30 min (TNFα) or 2 hrs (RANKL and perilipin-1) at room temperature, followed by overnight incubation at 4°C with anti-TNFα antibody (#ab6671, Abcam, Cambridge, United Kingdom) or anti-mouse perilipin-1 (#9349, Cell Signaling Technology, Danvers, MA, USA) (1:100) and anti-mouse RANKL (#sc-7628, Santa Cruz Biotechnology, Dallas, TX, USA) (1:50) in TNB blocking buffer. For RANKL and perilipin-1, after washing, slides were further incubated with Alexa flour 546 donkey anti-rabbit IgG (#A10040, Invitrogen, Carlsbad, CA, USA) (1:100) and Alexa flour 488 donkey anti-goat IgG (#A11055, Invitrogen) (1:100) in TNB blocking buffer for 1 hr at room temperature. For TNFα, after washing, slides were incubated with biotin-SP (long spacer)-conjugated AffiniPure goat anti-rabbit (#111-065-144, Jackson ImmunoResearch Laboratories, West Grove, PA, USA) for 30 min at room temperature. Subsequently, slides were washed and incubated for 30 min at room temperature with streptavidin-conjugated horseradish peroxidase (#43-8323, Zymed Laboratories, San Francisco, CA, USA) and tyramide following the manufacturer's protocol (TSA Biotin Kit, PerkinElmer). After washing, slides were incubated with streptavidin-conjugated Texas Red (#43-4317, Zymed Laboratories) for 30 min at room temperature. To reduce background, slides were incubated with TrueVIEW autofluorescence quenching solution (Vector Laboratories, Burlingame, CA, USA) for 2 min at room temperature for all immunofluorescent staining. Cell nuclei were counterstained with 5 μg/ml 4′, 6-diamidino-2-phenylindole (DAPI) (Thermo Fisher Science). Images were acquired under the fluorescence microscope with a 20× objective (BZ-X700, Keyence, Osaka, Japan) or a 40× objective (Zeiss, Jena, Germany). The number of RANKL-expressing adipocytes (defined as perilipin-1-expressing cells) in the marrow space within 300 μm from the proximal epiphyseal plate was counted using ImageJ.

Immunohistochemistry

Immunohistochemistry for 4-HNE was performed on deparaffinized sections of the L3 and L4 vertebrae from adult (4 months old) and middle-aged (13 months old) male mice. Endogenous peroxidase activity was inhibited by 3% H_2O_2 treatment for 10 min. Antigen retrieval was performed with Tris-EDTA buffer (10 mM Tris, 1 mM EDTA, 0.05% Tween 20, pH 9.0) in a boiling water bath at 95°C for 12 min. Slides were cooled to room temperature and blocked with TNB blocking buffer for 30 min at room temperature and incubated with anit-4-HNE antibody (#GTX40953, GeneTex, Irvine, CA, USA) in TNB blocking buffer overnight at 4°C. Slides were washed and incubated with rabbit anti-goat IgG (#305-065-003, Jackson ImmunoResearch Laboratories) for 30 min at room temperature. Subsequently, slides were washed and incubated for 30 min with streptavidin-conjugated horseradish peroxidase (#43-8323, Zymed Laboratories) and tyramide following the manufacturer's protocol (TSA Biotin Kit, PerkinElmer). The signal was developed with a 3,3'-diaminobenzidine (DAB) substrate-chromogen system (Vector Laboratories). Cell nuclei were counterstained with hematoxylin. Bright field images were acquired under the microscope with a 40× objective (Nikon Eclipse E800, Nikon, Tokyo, Japan) or with a 20× objective (EVOS XL, Thermo Fisher Scientific). For quantitative analysis, using ImageJ, 4-HNE positive osteocytes were counted with a 20× objective in three-five adjacent fields per vertebra and normalized to the total number of osteocytes per field.

Marrow adiposity analysis

Bone marrow adiposity was quantitatively analyzed, using ImageJ, as the ratio of adipocyte area over the marrow area within 300 or 1,200 µm from the proximal epiphyseal plate of the tibiae of middle-aged male (13 months old) or aging female (20 months old) animals, respectively. Briefly, bright-field images of H&E-stained proximal tibiae were acquired under the microscope with a 4x objective (Keyence). Using the segmented line tool (width = 300 or 1,200 µm) on ImageJ, the marrow area of interest was defined by tracing the epiphyseal plate manually. The area of marrow adipocytes (represented as white patches with a round or oval shape) was manually traced by using the polygon selection tool.

TUNEL assay

TUNEL staining was performed on deparaffinized tibiae sections of Dmp1-PPR^{KO} and control male (13 months old) and female (20 months old) mice following the manufacturer's protocol (DeadEnd colorimetric TUNEL, Promega, or TUNEL chromogenic apoptosis detection kit, GeneCopoeia, Rockville, MD, USA). For the TUNEL chromogenic kit (GeneCopoeia), modifications were made on the antigen retrieval and permeabilization step where the deparaffinized and rehydrated tibia sections were exposed to 10 mM citrate buffer (pH 6.0) for 5 min at 48°C and 0.05% pepsin (Roche, Basel, Switzerland) in 0.1 N HCl (Fisher Scientific) for 4 min at 37°C, respectively as reported previously [30]. Cell nuclei were counterstained with either hematoxylin or 0.5% methyl green (Sigma). Images were acquired under the microscope with a 40X objective (EVOS XL). The number of TUNEL-positive apoptotic osteocytes in the cortical and trabecular bone was counted using ImageJ either manually or unbiasedly. For the unbiased quantitative analysis, images were first color-deconvoluted into three single-colored images (methyl green, DAB, and background color) by manually selecting regions of interest that represent each color. The intensity of DAB signal on each osteocyte (identified as a methyl green positive cell within the bone matrix) was quantified and osteocytes with high and homogeneous DAB signal (threshold: mean >100, modal >60, standard deviation >60 per cell) were defined as TUNEL-positive.

Serology

Serum was isolated from the blood collected by retro-orbital bleeding or cardiac puncture using pre-heparinized capillary tubes (Fisher Scientific) or insulin syringes (BD) heparinized with 1000 IU/ml of heparin (Alfa Aesar), respectively. Serum levels of P1NP (Rat/Mouse P1NP enzyme immunoassays (EIA)) and CTX (RatLaps EIA) were measured using ELISA (Immunodiagnostic Systems, Tyne and Wear, United Kingdom). Serum calcium was measured by calcium liquicolor arsenazo kit (Stanbio Laboratory, Boerne, TX, USA) and inorganic phosphate was quantified by phospho liquid-UV kit (Stanbio Laboratory) according to the manufacturer's instructions. Serum PTH was measured using mouse intact PTH ELISA kit (MicroVue Bone Mouse PTH 1-84, Quidel Corporation, San Diego, CA, USA) according to the manufacturer's protocol. Serum sclerostin was measured using mouse/rat SOST/Sclerostin Quantikine ELISA kit (#MSST00, R&D Systems, Minneapolis, MN, USA) according to the manufacturer's protocol. Serum cytokines included IL-6, -10, and -12p70, MCP-1, interferon γ, and TNF were measured using BD cytometric bead array mouse inflammation kit (BD) following the manufacturer's instructions.

µCT analysis

The left femora and the L5 vertebrae of adult (4 months old) and middle-aged (13–16 months old) male mice were isolated and cleaned of soft tissues. The femora were either fixed in 70% ethanol at 4°C or frozen at –20°C being wrapped by PBS-soaked gauze (8-ply, Covidien, Dublin, Ireland). The L5 vertebrae were fixed in 10% phosphate-buffered formalin (pH 6.9–7.2) for at least 48 hrs and stored in 70% ethanol. Bone morphology and microarchitecture were analyzed in the distal femoral metaphysic, the femoral midshaft, and the vertebral body using a desktop high-resolution µCT (µCT40, Scanco Medical, Wangen-Brüttisellen, Switzerland), as described previously [22]. Following parameters were assessed for the trabecular bone region: bone volume over total tissue volume, BV/TV (%); trabecular number, Tb.N (/mm); trabecular thickness, Tb.Th (mm); trabecular separation, Tb.Sp (mm); and for the cortical bone region: cortical thickness, Cort.Th (mm); cortical density, Cort.Dens (mmHA/ccm); cortical area, Cort.A (mm^2); medullary area, MA (mm^2); cortical porosity, Cort.Por (%); cortical density, Cort.Den (mgHA/ccm); polar moment of inertia, pMOI (mm^4). See Supplementary Table 1 for the full list of analyzed parameters.

Histomorphometric analysis

Adult (4 months old) and middle-aged (13 months old) male mice were injected intraperitoneally with 20 mg/kg calcein (Sigma) on days 9 and 2 before sacrifice. The left femora and the L5 vertebrae were isolated, cleaned of adherent tissues, and used for µCT analysis (described above) prior to histomorphometric analysis. Both the femora and vertebrae were dehydrated, infiltrated, and embedded in methyl methacrylate.

Undecalcified 4-μm-thick sections were cut using a microtome (Leica RM 2255, Heidelberg, Germany). Consecutive sections were stained by von Kossa with van Gieson counterstain and toluidine blue to determine structural and cellular parameters, respectively. Dynamic parameters were quantified using unstained and undecalcified sections of the femora. Undecalcified sections of the femora were also stained for TRAP. Histomorphometric parameters (i.e., formation and resorption) were measured using the Osteomeasure image analysis system (OsteoMetrics, Decatur, GA, USA) coupled to a microscope (BX 50 combined with a DP72 digital camera, Olympus, Hamburg, Germany). A sampling site of about 2 mm^2 was established in the cancellous bone at 400 μm below the growth plate. Analysis was done in a standardized fashion by an experienced scientist and results were expressed according to the updated standardized American Society for Bone and Mineral Research (ASBMR) nomenclature [4]. In detail, cancellous bone volume in the distal femora and the L5 vertebrae was assessed as BV/TV (%). Tb.Th, Tb.N, and Tb.Sp were also calculated both in the distal femora and in the L5 vertebrae. Additional parameters included: mineralizing surface over bone surface, MS/BS (%); mineral apposition rate, MAR (μm/day); bone formation rate (BFR) over bone surface, BFR/BS; over bone volume, BFR/BV (%/year); and over total volume, BFR/TV (%/year); osteoid volume (OV) over bone volume, OV/BV (%); and osteoid surface (OS) over bone surface, OS/BS (%). Formation parameters were expressed as osteoblast surface per bone surface, Ob.S/BS (%); osteoblast number per bone perimeter, N.Ob/B.Pm (/mm); and osteoblast number per tissue area, N.Ob/T.Ar (/mm^2). Resorption parameters were expressed as osteoclast surface per bone surface, Oc.S/BS (%); osteoclast number per bone perimeter, N.Oc/B.Pm (/mm); and osteoclast number per tissue area, N.Oc/T.Ar (/mm^2). We also quantified the number of osteocytes per bone volume, N.Ot/BV (/mm^2), in the cancellous bone. Cortical bone parameters, such as Cort.Th (mm); endocortical mineral apposition rate, End.Cort MAR (μm/day); and osteocyte density, Ot density (/mm^2), were analyzed at the femoral midshaft. See Supplementary Table 1 for the full list of analyzed parameters.

RNA isolation and purification

The right femora and/or tibiae were dissected from adult male (4 months old) and middle-aged (13–16 months old) male or middle-aged and aging (13 and 20 months old) female mice. The bones were cut at both proximal and distal end, centrifuged briefly at 8.0–9.0 × g for 7 sec (at start) to remove bone marrow, placed in 1 ml of TRIzol reagent (Thermo Fisher Scientific, Waltham, MA, USA) with a grinding stainless steel bead (5-mm diameter, OPS Diagnostics, Lebanon, NJ, USA), snap-frozen in liquid nitrogen, and subsequently stored at –80°C for RNA isolation. The bone marrow removed from the femora and/or tibiae was lysed in 600 μl of lysis buffer (RLT Plus (Qiagen) supplemented with 1% β-mercaptoethanol (Sigma)) by pipetting and stored at –80°C for RNA isolation. Cells under *in vitro* culture were harvested in 350 μl of lysis buffer and either frozen at –80°C or directly processed for RNA isolation.

Prior to RNA isolation, the frozen bones were homogenized with TissueLyser II (Qiagen) at 30 Hz at room temperature for 8–12 min (including the time for thawing), refrozen in liquid nitrogen, and subsequently restored at –80°C. Bone marrow and *in vitro* cell samples were both homogenized with QIAshredder (Qiagen). The total RNA from long bones or bone marrow and *in vitro* cells was isolated using PureLink RNA mini kit (Invitrogen) or RNeasy Plus mini kit (Qiagen), respectively, as per manufacturer's instructions. Some of the bones from middle-aged male (13 months old) mice were subjected to sequential collagenase (Worthington Biochemical, Lakewood, NJ, USA) and EDTA digestions to remove endosteal and periosteal osteoblasts and bone marrow cells and snap-frozen in liquid nitrogen followed by homogenization and RNA isolation. The quality and quantity of the total RNA was measured by UV spectrophotometry (NanoDrop 2000c, Thermo Fisher Scientific).

Quantitative real time PCR

RNA was reverse transcribed into complementary DNA (cDNA) using PrimeScript RT reagent kit (Clontech, Takara Bio, Kusatsu, Shiga, Japan) with a genomic DNA elimination step or qScript cDNA SuperMix (QuantaBio, Beverly, MA, USA). Real time qPCR assay was subsequently performed with StepOne Plus (Applied Biosystems) using SYBR Green (Power SYBR, Thermo Fisher Scientific). The PCR conditions were as follows: one cycle of 95°C (10 min), 50 cycles of 95°C, 58°C, and 72°C (15 sec, 15 sec, and 30 sec, respectively), and one cycle of 95°C (30 s), followed by a melting curve step (58°C to 95°C with an increment of +0.5°C, 1 min for each temperature). See Supplementary Table 2 for the primer sequences. Data analysis was based on the comparative cycle threshold (ΔCT) method and expression of each gene was normalized to *Actb* expression or the geometric mean of the three gene expression (Actb, *B2m*, and *Gapdh*), which were selected as the most stable housekeeping genes among the eight genes (*Actb*, *B2m*, *Gapdh*, *Hprt*, *Ywhaz*, *Rlp13a*, *Sdha*, and 18S) by geNorm (qbase+ software, Biogazelle, Gent, Belgium) [5].

Treatment of BMSCs with OEBE conditioned medium or mouse serum

BMSCs were isolated from control male mice (3–4 months of age) and incubated with either 50% (v/v) of conditioned medium from OEBEs, as previously described [39], or 3% (v/v) mouse serum (along with 7% (v/v) FBS) of 13 months old control and Dmp1-PPR^{KO} mice for 6 or 4 days under osteogenic or adipogenic differentiation medium, respectively. For ALP activity assay, cells were lysed with 0.1% Triton X-100 in Tris buffer (pH 7.6) and the cell extract (25 µl) was incubated with 25 µl mix of 2-aminomethyl propanol (0.5 M) (Sigma), $MgCl_2$ (5.3 mM) (Sigma) and Na-p-nitrophenyl phosphate (1.3 mM) (Sigma) for 10 min at 37°C. The reaction was stopped by NaOH (0.2 N) (Fisher Scientific) and the absorbance was measured at 405 nm using a spectrophotometer (TECAN, Mannedorf, Switzerland). ALP activity was normalized by protein content measured by Bradford method using protein assay dye reagent concentrate (Bio-Rad, Hercules, CA, USA). Cell proliferation was measured using PrestoBlue cell viability reagent (Invitrogen) as per manufacturer's protocol. For adipogenic differentiation assay, BMSCs were cultured in differentiation medium (αMEM containing 0.5 mM 3-Isobutyl-1-methylxanthine (IBMX) (Acros Organics), 1 µM dexamethasone (Sigma), 10 µg/ml insulin (Sigma), and 1 µM rosiglitazone (Sigma)) for 2 days at 37°C + 5% CO_2 in the presence of the mouse serum. Cells were then maintained in base medium (αMEM containing 10 µg/ml insulin and 1 µM rosiglitazone) for additional 2 days in the presence of the mouse serum. Cells were fixed with 10% phosphate-buffered formalin for 15 min followed by incubation with 0.3% oil-red O (Sigma) for 30 min at 37°C. Bright field images were acquired under the microscope with a 10X objective (Keyence). Quantification of lipid was performed by elution of oil-red O stain and by measuring absorbance at 540 nm using a spectrophotometer (Berthold Technologies).

TRAP staining and activity assay

Histology sections of tibiae and/or femora were deparaffinized, rehydrated, and stained for TRAP as described previously [6]. Briefly, the sections were incubated in acetate buffer (0.2 M sodium acetate and 50 mM sodium tartrate, pH 5.0) for 20 min at room temperature followed by a 30 min incubation in the same buffer containing 0.5 mg/ml naphthol AS-MX phosphate and 1.1 mg/ml Fast Red Violet LB Salt (#F3381, Sigma) at 37°C. For *in vitro* experiments, BMMCs were isolated from control mice (3–4 months of age) and purified by Ficoll-Paque (GE Healthcare, Chicago, IL, USA) purification as described previously [39]. BMMCs were cultured for 3 days with 25 ng/ml M-CSF (Shenandoah Biotechnology, Warwick, PA, USA) followed by 4 days of incubation with 3% (v/v) mouse serum (along with 7% (v/v) FBS) from control and Dmp1-PPR^{KO} mice in the presence of 25 ng/ml M-CSF and 50 ng/ml RANKL (Shenandoah Biotechnology). Medium was replaced every 3 days. Cells were then fixed with 10% formalin and incubated in TRAP staining buffer (the acetate buffer containing 0.1 mg/ml naphthol AS-MX phosphate and 0.6 mg/ml Fast Red Violet LB Salt) for 10 min at 37°C. Cell images were acquired by the microscope with a 4X objective (Keyence) and the number and size of TRAP+ multinuclear osteoclasts (>3 nuclei) was quantified using ImageJ. For TRAP activity assay, conditioned medium (30 µl) was incubated with TRAP staining buffer (170 µl) for 2 hrs at 37°C and absorbance was measured at 590 nm using a spectrophotometer (Berthold Technologies).

Cyclic AMP assay

Control and 12H-PPR^{KO} cells were plated on 96-well plate at a density of 5×10^3 cells/well. After 4–5 days of culture at 33°C, the cells were treated with 10 nM hPTH(1–34) or 10 µM FSK for 30 min at room temperature under 3-Isobutyl-1-methylxanthine (IBMX) buffer (0.5 mg/ml IBMX (Acros Organics), 10% BSA, 1 M HEPES dissolved in Hank's balanced salt solution (Sigma)). Reaction was stopped by adding HCl (16.6 mM) (Fisher Scientific) and the cells were frozen immediately at –80°C for RIA analysis.

REFERENCES

1. Ran FA, Hsu PD, Wright J, Agarwala V, Scott DA, Zhang F. Genome engineering using the CRISPR-Cas9 system. Nat Protoc. 2013; 8:2281–308. https://doi.org/10.1038/nprot.2013.143 PMID:24157548

2. Sinha P, Aarnisalo P, Chubb R, Ono N, Fulzele K, Selig M, Saeed H, Chen M, Weinstein LS, Pajevic PD, Kronenberg HM, Wu JY. Loss of Gsα early in the osteoblast lineage favors adipogenic differentiation of mesenchymal progenitors and committed osteoblast precursors. J Bone Miner Res. 2014; 29:2414–26. https://doi.org/10.1002/jbmr.2270 PMID:24806274

3. Morimoto D, Kuroda S, Kizawa T, Nomura K, Higuchi C, Yoshikawa H, Tomita T. Equivalent osteoblastic differentiation function of human mesenchymal stem cells from rheumatoid arthritis in comparison with osteoarthritis. Rheumatology (Oxford). 2009; 48:643–9. https://doi.org/10.1093/rheumatology/kep044 PMID:19398485

4. Dempster DW, Compston JE, Drezner MK, Glorieux FH, Kanis JA, Malluche H, Meunier PJ, Ott SM, Recker RR, Parfitt AM. Standardized nomenclature, symbols, and units for bone histomorphometry: a 2012 update of the report of the ASBMR Histomorphometry Nomenclature Committee. J Bone Miner Res. 2013; 28:2–17.
https://doi.org/10.1002/jbmr.1805
PMID:23197339

5. Vandesompele J, De Preter K, Pattyn F, Poppe B, Van Roy N, De Paepe A, Speleman F. Accurate normalization of real-time quantitative RT-PCR data by geometric averaging of multiple internal control genes. Genome Biol. 2002; 3:RESEARCH0034.
https://doi.org/10.1186/gb-2002-3-7-research0034
PMID:12184808

6. Erlebacher A, Derynck R. Increased expression of TGF-beta 2 in osteoblasts results in an osteoporosis-like phenotype. J Cell Biol. 1996; 132:195–210.
https://doi.org/10.1083/jcb.132.1.195
PMID:8567723
