# Supplementary material for: Parathyroid hormone signaling in mature osteoblasts/osteocytes protects mice from age-related bone loss
Source: Aging (Albany NY). 2021 Dec 30;13(24):25607–42. doi: 10.18632/aging.203808 (PMC8751595; doi:10.18632/aging.203808)
Supplement: Supplementary Figures [file aging-13-203808-s002.pdf]

SUPPLEMENTARY FIGURES

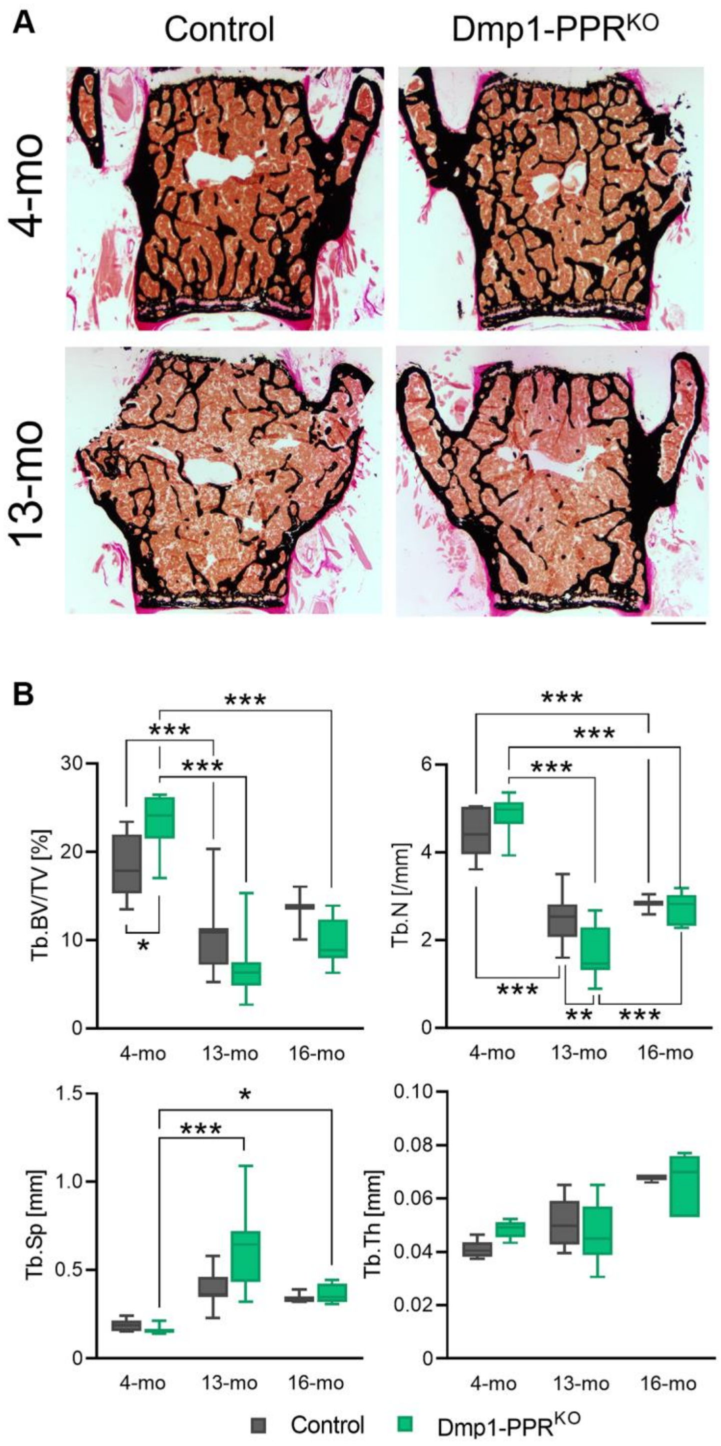

Supplementary Figure 1. Analysis of the skeletal phenotype of Dmp1-PPR^{KO} mice. (**A**) Representative images of Von Kossa staining on the L5 vertebrae of 4- and 13-month-old male control and Dmp1-PPR^{KO} mice. (**B**) Skeletal parameters of the distal femora analyzed by μCT were compared among male control and Dmp1-PPR^{KO} animals at different ages (4, 13, or 16 months of age). Data is shown as box and whisker plot. *N* = 7-15 per group. Two-way ANOVA with Tukey's *post hoc* test was performed. $^{*}p < 0.05$, $^{**}p < 0.01$, $^{***}p < 0.001$.

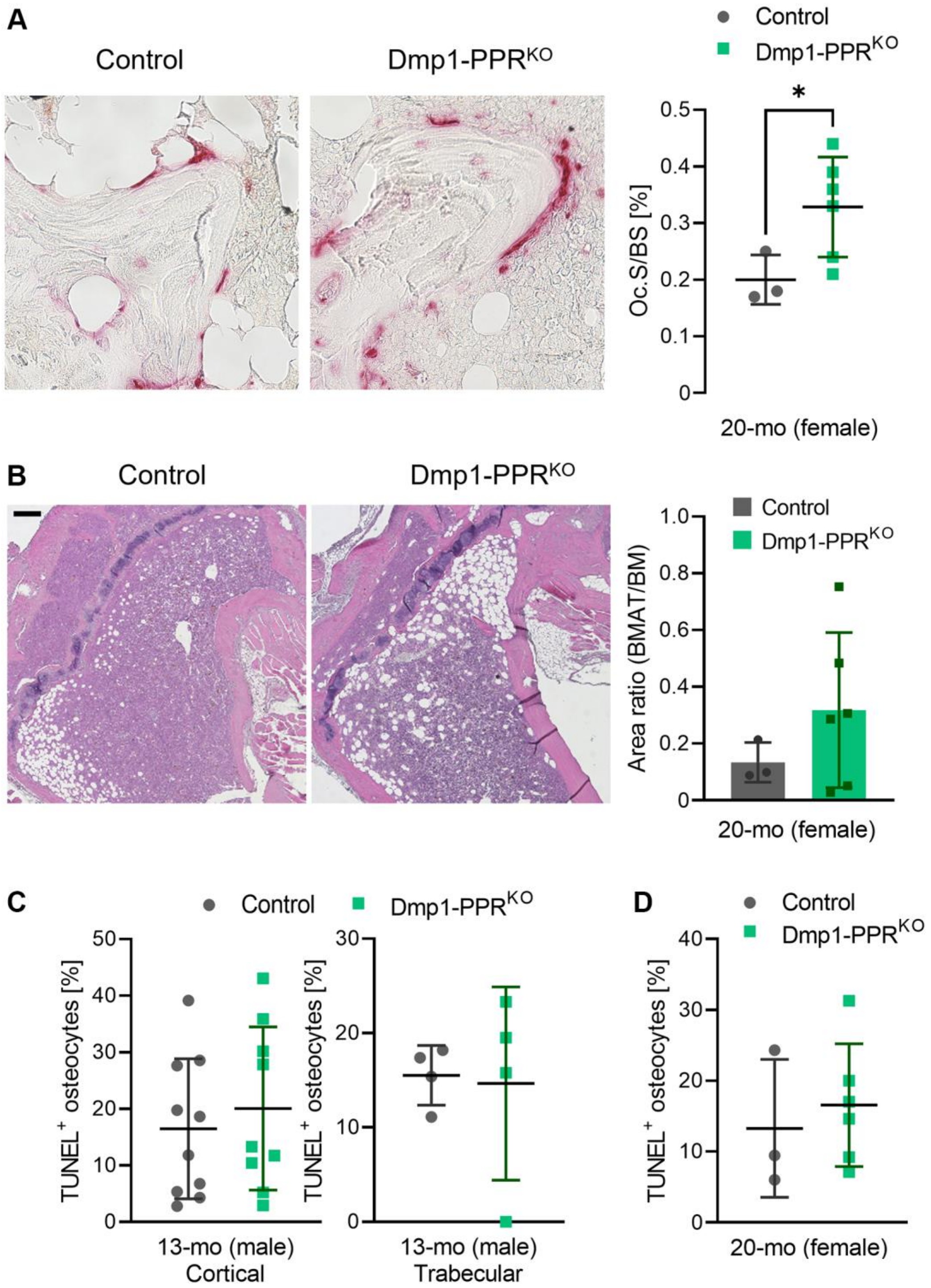

Supplementary Figure 2. (**A**) Representative images of TRAP staining and quantification of Oc.S/BS on the proximal tibiae (trabecular compartment) of female control and Dmp1-PPR^{KO} mice at 20 months of age are shown. *N* = 3–6 per group. (**B**) Representative images of H&E staining and quantification of BMAT/BM ratio on the proximal tibiae of female control and Dmp1-PPR^{KO} mice at 20 months old are shown. *N* = 3–6 per group. (**C**, **D**) TUNEL analysis on the tibiae from aging control and Dmp1-PPR^{KO} mice. (**C**) Quantification of TUNEL+ osteocytes in the tibiae of 13-month-old male control and Dmp1-PPR^{KO} mice is shown. Analysis was performed on the cortical region (left, midshaft) and the trabecular region (right, proximal) of the tibiae. *N* = 4–10 per group. (**D**) Quantification of TUNEL+ osteocytes in the tibiae (cortical region) of 20-month-old female control and Dmp1-PPR^{KO} mice is shown. *N* = 3–6 per group. Data are presented as mean ± SD. Unpaired student's or Welch's *t* test was performed. $^{*}p < 0.05$.

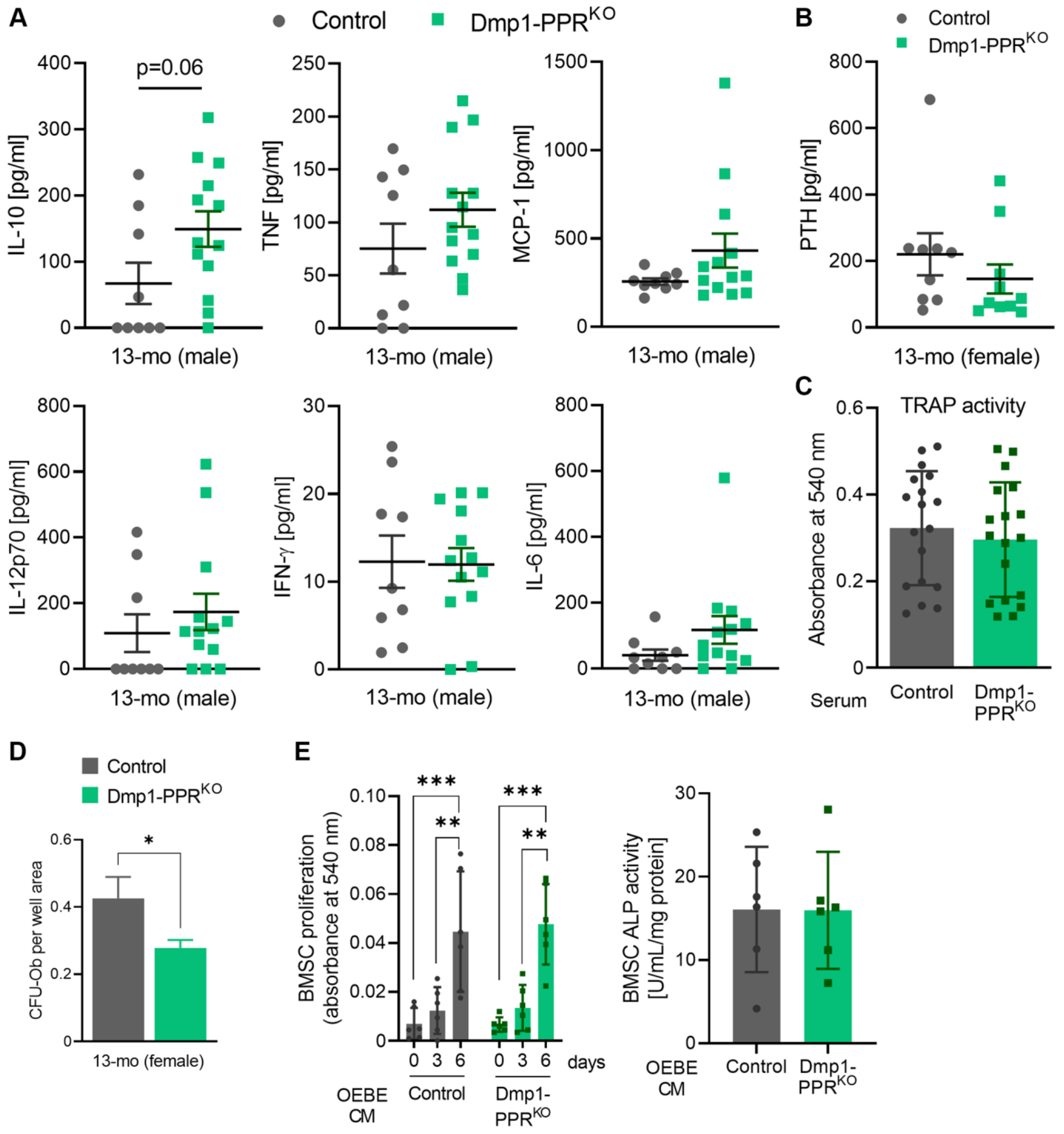

Supplementary Figure 3. (**A**) Serum cytokines in 13-month-old Dmp1-PPR^{KO} mice were analyzed. Quantification of serum cytokines of 13-month-old male control and Dmp1-PPR^{KO} mice is shown. Data are expressed as mean ± SEM. *N* = 7–13 per group. (**B**) Serum PTH levels in 13-month-old female mice were analyzed by ELISA. Means ± SEM are shown. (**C**) TRAP activity was measured in the conditioned medium from BMMCs under osteoclastic differentiation in the presence of control or Dmp1-PPR^{KO} serum. Means ± SD are shown. (**D**) CFU-Ob assay was performed on BMSCs isolated from female control and Dmp1-PPR^{KO} mice at 13 months of old is shown. Means ± SEM are shown. (**E**) Proliferation (left) and ALP activity measured on BMSCs under osteogenic differentiation in the presence of conditioned medium from control and Dmp1-PPR^{KO} OEBEs culture. Means ± SD are shown. Unpaired student's *t* test was performed. $^{*}p < 0.05$, $^{**}p < 0.01$, $^{***}p < 0.001$.

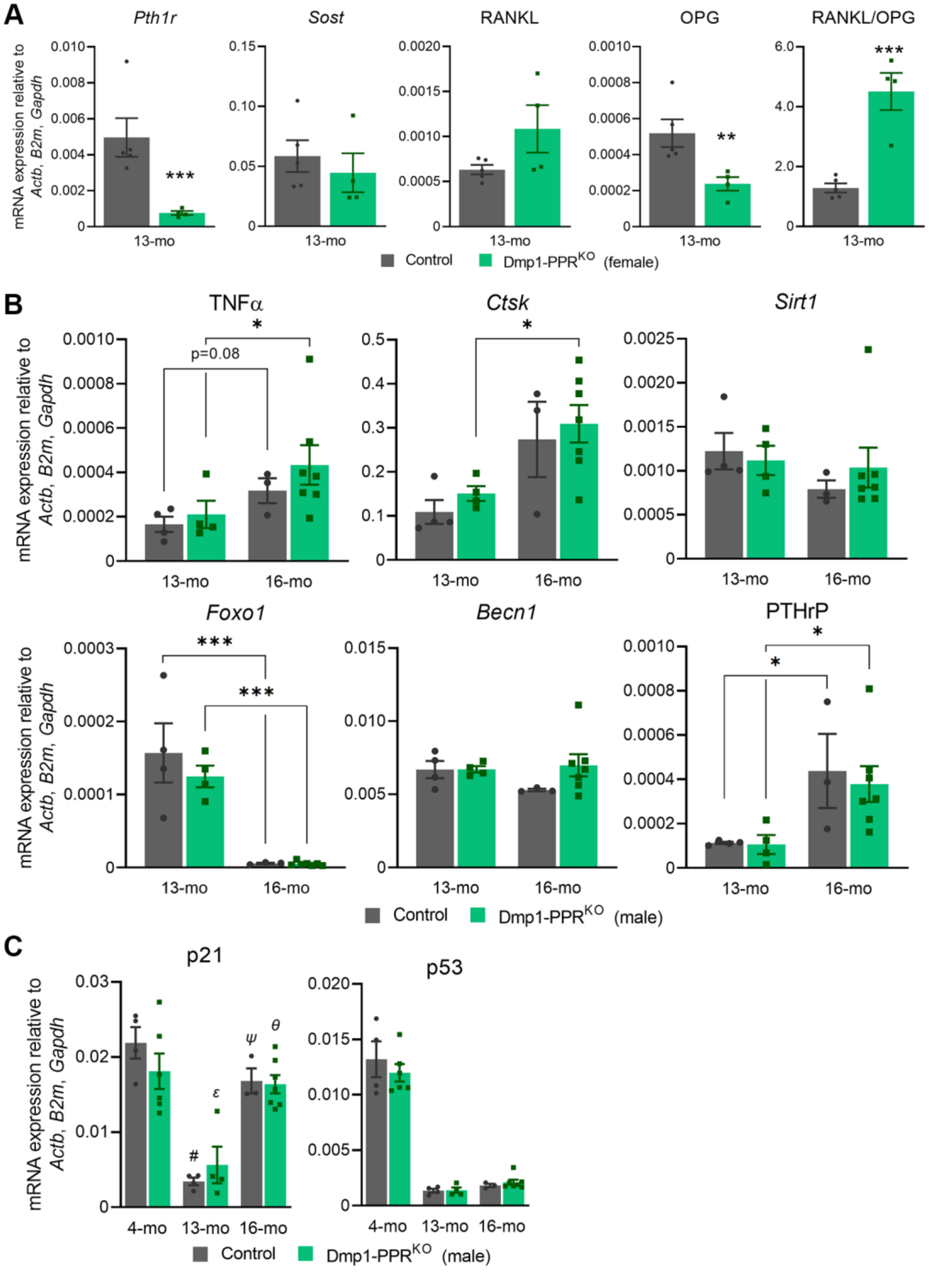

Supplementary Figure 4. (**A**) Gene expression in marrow-removed femora from 13-month-old female mice is shown. *N* = 4–5 per group. (**B**, **C**) Gene expression in the tibiae and/or femora of control and Dmp1-PPR^{KO} mice in male (4, 13, and 16 months of age). Expression of genes involved in osteoclasts, autophagy and/or senescence was analyzed by qPCR. *N* = 4–11 per group. Data are presented as mean ± SEM. One-way ANOVA with Sidak's *post hoc* test, Two-way ANOVA with Tukey's *post hoc* test or unpaired student's *t* test was performed. $^{*}p < 0.05$, $^{***}p < 0.001$, $^{\#}p < 0.001$ (vs. 4-mo Control), $^{\varepsilon}p < 0.001$ (vs. 4-mo Dmp1-PPR^{KO}), $^{\psi}p < 0.001$ (vs. 13-mo Control), $^{\theta}p < 0.001$ (vs. 13-mo Dmp1-PPR^{KO}).

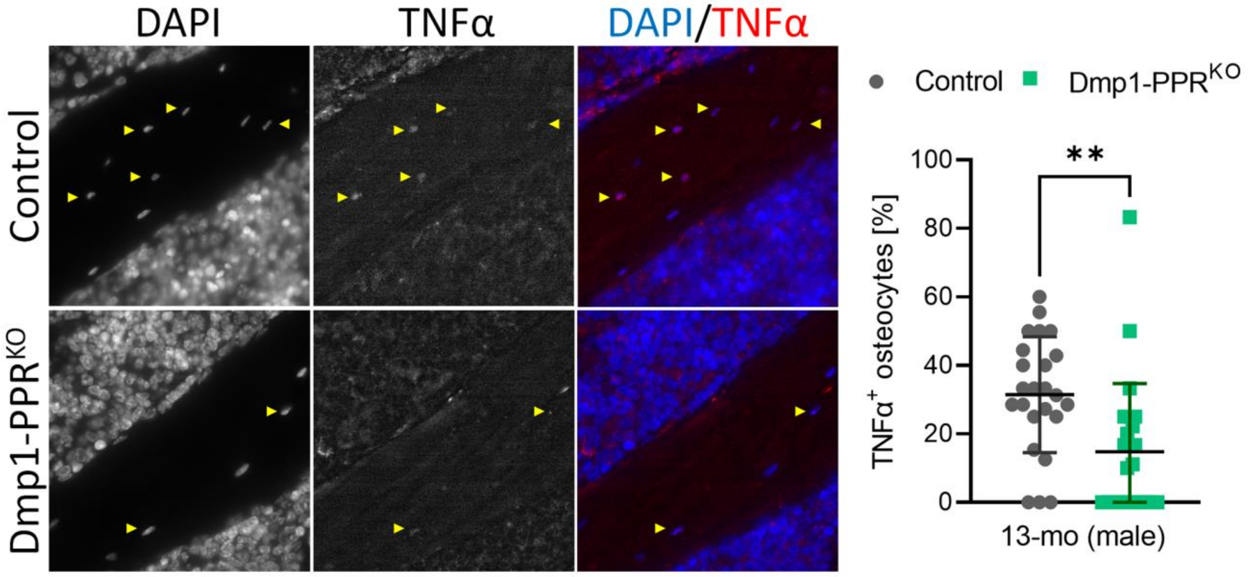

Supplementary Figure 5. Representative TNFα immunofluorescent images of the tibia of 13-month-old male mice. The frequency of TNFα+ (red) osteocytes (yellow arrowheads) per field was quantified over the total number of osteocytes (stained with DAPI, blue). *N* = 4 per group. Data are presented as mean ± SD. Unpaired Welch's *t* test was performed. $^{**}p < 0.01$.

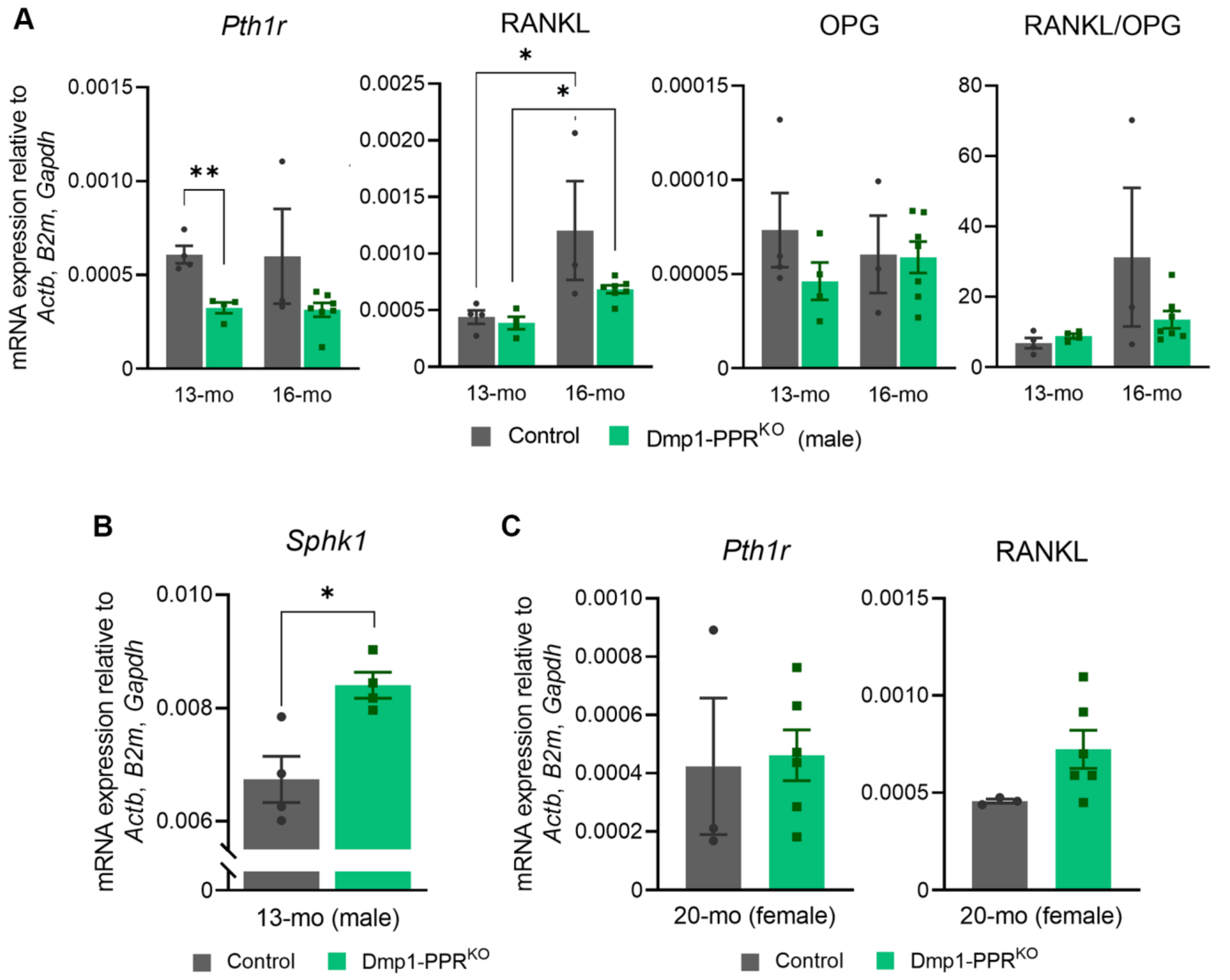

Supplementary Figure 6. Gene expression in bone marrow cells of control and Dmp1-PPR^{KO} mice. (**A**) Expression of PPR, RANKL, OPG and RANKL/OPG ratio in bone marrow from middle-aged male control and Dmp1-PPR^{KO} mice (13 and 16 months old) was analyzed by qPCR. *N* = 3–11 per group. (**B**) Expression of Sphk1 in the bone marrow isolated from the femora of middle-aged male animals (13-month-old) was analyzed by qPCR. *N* = 4 per group. (**C**) Expression of PPR and RANKL in bone marrow from female control and Dmp1-PPR^{KO} mice (20 months old) was analyzed by qPCR. *N* = 3–6 per group. Data are presented as mean ± SEM. Unpaired Student's *t* test was performed. $^{*}p < 0.05$, $^{**}p < 0.01$.

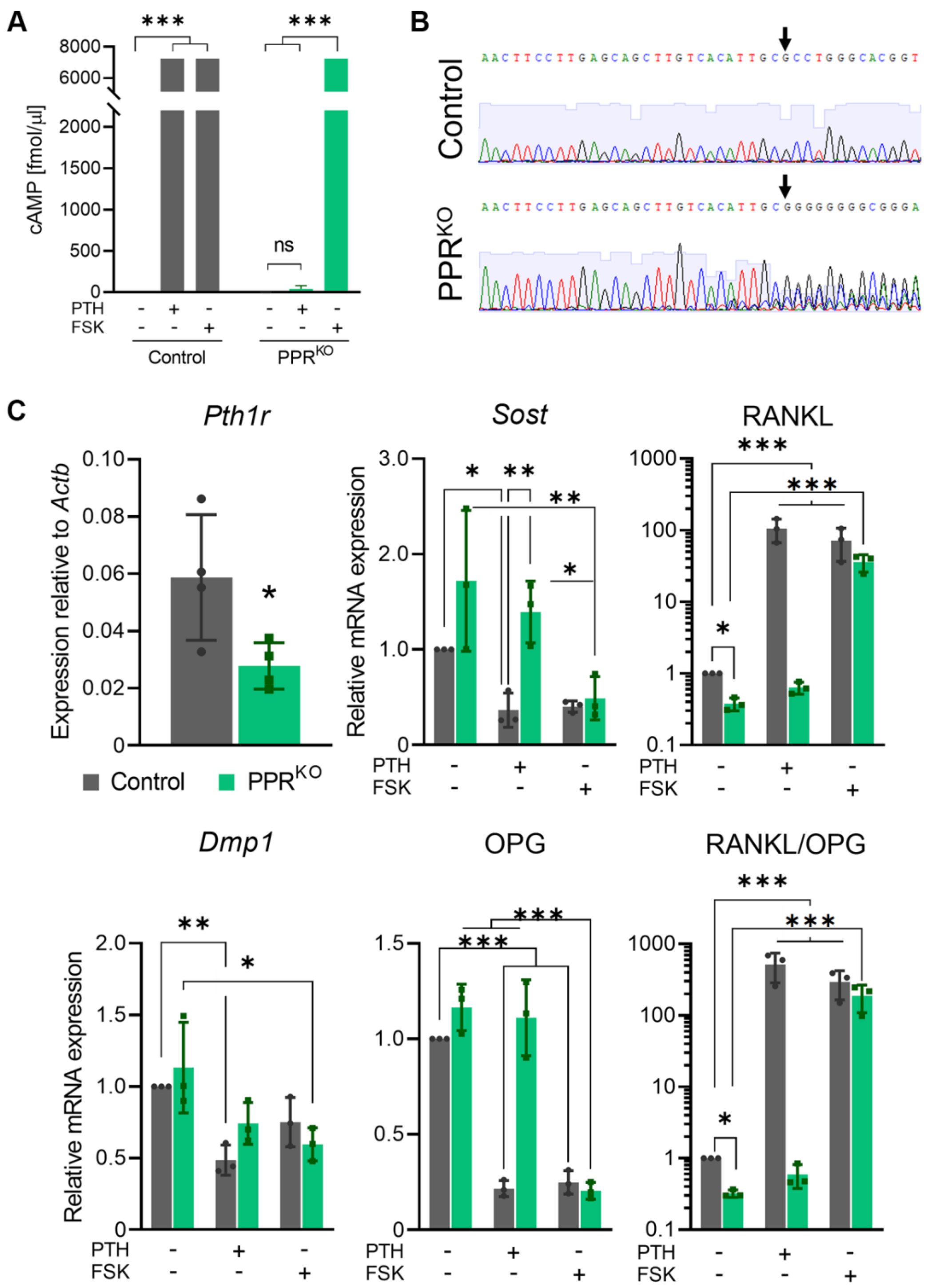

Supplementary Figure 7. Characterization of Ocy454-12H PPR^{KO} cells. (**A**) cAMP accumulation assay in 10 nM PTH- or 10 µM forskolin-treated control and PPR^{KO} cells. PPR^{KO} cells showed no cAMP response to PTH but forskolin. (**B**) Sanger DNA sequencing results of the targeted Pth1r exon 3 sequence in control and PPR^{KO} cells are shown. Black arrows indicate the cleavage site by Cas9 protein. Unlike control cells, the sequence of PPR^{KO} after the cleavage site was unreadable due to random repair via nonhomologous end joining (NHEJ). (**C**) Expression of PPR and osteocytic genes in control and PPR^{KO} cells treated with 10 nM PTH or 10 µM forskolin was analyzed by qPCR. N = 3 per group. Data are presented as mean ± SD. Two-way ANOVA with Tukey's *post hoc* test was performed. $^{*}p < 0.05$, $^{**}p < 0.01$, $^{***}p < 0.001$.

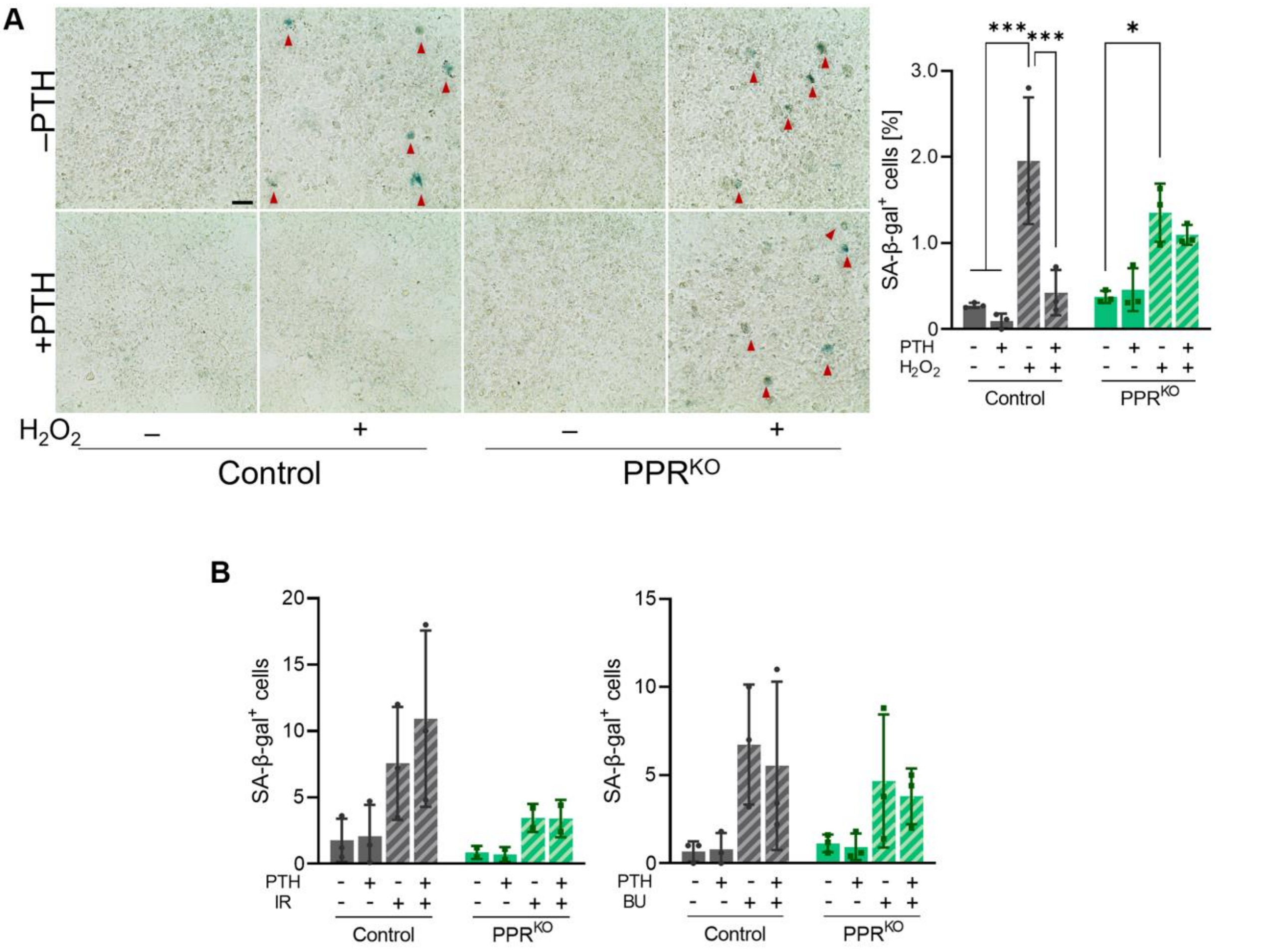

Supplementary Figure 8. (**A**) SA β-gal staining on Ocy454-12H PPR^{KO} under oxidative stress. Cells were pretreated with 10 nM hPTH(1–34) for 18-22 hrs and then exposed to H_2O_2 (150 μM, 7 days). (**B**) SA β-gal staining on irradiated (5 Gy) or busulfan (50 μM)-treated control and PPR^{KO} cells. *N* = 3 per group. Data are presented as mean ± SD. Two-way ANOVA with Tukey's *post hoc* test was performed. $^{*}p < 0.05$, $^{***}p < 0.001$.
