# Supplementary material for: Parathyroid hormone signaling in mature osteoblasts/osteocytes protects mice from age-related bone loss
Source: Aging (Albany NY). 2021 Dec 30;13(24):25607–42. doi: 10.18632/aging.203808 (PMC8751595; doi:10.18632/aging.203808)
Supplement: Supplementary Tables [file aging-13-203808-s003.pdf]

SUPPLEMENTARY TABLES

Supplementary Table 1. Abbreviations of bone parameters for histomorphometrical and μCT analysis.

Type of index	Parameter	Abbreviation	Unit	Analyzed bone
Structural	Bone volume/tissue volume	BV/TV	%	Distal femur, L5 vertebra
(Trabecular)	Number	Tb.N	/mm	
	Thickness	Tb.Th	mm	
	Separation	Tb.Sp	mm	
Structural	Thickness	Cort.Th	mm	Midshaft femur
(Cortical)	Density	Cort.Dens	mmHA/ccm	
	Area	Cort.A	mm^2	
	Medullary area	MA	mm^2	
	Porosity	Cort.Por	%	
	Polar moment of inertia	pMOI	mm^4	
Dynamic	Mineralizing surface/bone surface	MS/BS	%	Distal and midshaft femur
	Mineral apposition rate	MAR	μm/day	
	Bone formation rate/bone surface	BFR/BS		
	Bone formation rate/bone volume	BFR/BV (%/year)	%/year	
Formation	Osteoid volume/bone volume	OV/BV	%	Distal femur
	Osteoid surface/bone surface	OS/BS	%	
	Osteoblast surface/bone surface	Ob.S/BS	%	
	Osteoblast number/tissue area	N.Ob/T.Ar		
	Osteoblast number/bone perimeter	N.Ob/B.Pm	/mm	
Resorption	Erosion surface/bone surface	ES/BS	%	Distal femur
	Osteoclast surface/bone surface	Oc.S/BS	%	
	Osteoclast number/bone perimeter	N.Oc/B.Pm	/mm	
Osteocyte	Osteocyte number/bone volume	N.Ot/BV	$/mm^2$	Distal and midshaft femur
		Ot density ($/mm^2$)	$/mm^2$	

Supplementary Table 2. Primer sequences for real time qPCR.

Gene Symbol	Forward (5′–3′)	Reverse (5′–3′)
18S	TCAAGAACGAAAGTCGGAGG	GGACATCTAAGGGCATCAC
Actb (β-actin)	GGCTGTATTCCCCTCCATCG	CCAGTTGGTAACAATGCCATGT
B2m	TTCACCCCCACTGAGACTGAT	GTCTTGGGCTCGGCCATA
Becn1	GAGGCTAACTCAGGAGAGGAGC	TGCCTCCCCGATCAGAGTGA
Bglap (Osteocalcin)	CCGGGAGCAGTGTGAGCTTA	TAGATGCGTTTGTAGGCGGTC
Cdkn1a ($p21^{Cip1}$)	CCTGGTGATGTCCGACCTG	CCATGAGCGCATCGCAATC
Cdkn2a ($p16^{Ink4a}$)	GAACTCTTTCGGTCGTACCC	AGTTCGAATCTGCACCGTAGT
Csf1 (M-CSF)	GACCCTCGAGTCAACAGAGC	TGTCAGTCTCTGCCTGGATG
Ctsk	GAAGAAGACTCACCAGAAGCAG	TCCAGGTTATGGGCAGAGATT
Dmp1	AAGCTAGCCCAGAGGGACAGGCAA	TTATCGGCGCCGGTCCCCGTAC
Foxo1	CCCAGGCCGGAGTTTAACC	GTTGCTCATAAAGTCGGTGCT

Gapdh	AGGTCGGTGTGAACGGATTTG	TGTAGACCATGTAGTTGAGGTCA
Hprt	TCAGTCAACGGGGGACATAAA	GGGGCTGTACTGCTTAACCAG
Pth1r (PPR)	CAGATTTTCCTGCTGCACCG	CTGCTGTGTGCAGAACTTCC
Pthlh (PTHrP)	CAGTTAGAGGCGCTGATTCC	AGCTCTGATTTCGGCTGTGT
Rlp13a	GGGCAGGTTCTGGTATTGGAT	GGCTCGGAAATGGTAGGGG
Sdha	GGAACACTCCAAAAACAGACCT	CCACCACTGGGTATTGAGTAGAA
Sirt1	GCTGACGACTTCGACGACG	TCGGTCAACAGGAGGTTGTCT
Sost (Sclerostin)	CTTCAGGAATGATGCCACAGAGGT	ATCTTTGGCGTCATAGGGATGGTG
Sphk1	GAACCATAACTCTGTGCCTTTGTCT	AGCAATGGGGAGTGTCTTCTATATG
Tnf (TNFα)	AGACCCTCACACTCAGATCATCTTC	CCACTTGGTGGTTTGCTACGA
Tnfsf11 (RANKL)	CACAGCGCTTCTCAGGAGCTC	GAGATCTTGGCCCAGCCTCGA
Tnfrsf11b (OPG)	ACCCAGAAACTGGTCATCAGC	CTGCAATACACACACTCATCACT
Trp53 (p53)	CTCTCCCCCGCAAAAGAAAAA	CGGAACATCTCGAAGCGTTTA
Ywhaz	GAAAAGTTCTTGATCCCCAATGC	TGTGACTGGTCCACAATTCCTT
